# Spatiotemporal modelling and mapping of cervical cancer incidence among HIV positive women in South Africa: a nationwide study

**DOI:** 10.1186/s12942-021-00283-z

**Published:** 2021-06-29

**Authors:** Dhokotera Tafadzwa, Riou Julien, Bartels Lina, Rohner Eliane, Chammartin Frederique, Johnson Leigh, Singh Elvira, Olago Victor, Sengayi-Muchengeti Mazvita, Egger Matthias, Bohlius Julia, Konstantinoudis Garyfallos

**Affiliations:** 1 Institute of Social and Preventive Medicine (ISPM), University of Bern, Bern, Switzerland; 2 National Cancer Registry, National Health Laboratory Service, Johannesburg, South Africa; 3 Graduate School for Cellular and Biomedical Sciences, University of Bern, Bern, Switzerland; 4 Centre for Infectious Disease Epidemiology and Research, School of Public Health and Family Medicine, University of Cape Town, Cape Town, South Africa; 5 Division of Epidemiology and Biostatistics, School of Public Health, University of the Witwatersrand, Johannesburg, South Africa; 6 Population Health Sciences, Bristol Medical School, University of Bristol, Bristol, UK; 7 Epidemiology and Biostatistics Department, School of Public Health, Imperial College London, London, UK

## Abstract

**Background:**

Disparities in invasive cervical cancer (ICC) incidence exist globally, particularly in HIV positive women who are at elevated risk compared to HIV negative women. We aimed to determine the spatial, temporal, and spatiotemporal incidence of ICC and the potential risk factors among HIV positive women in South Africa.

**Methods:**

We included ICC cases in women diagnosed with HIV from the South African HIV cancer match study during 2004–2014. We used the Thembisa model, a mathematical model of the South African HIV epidemic to estimate women diagnosed with HIV per municipality, age group and calendar year. We fitted Bayesian hierarchical models, using a reparameterization of the Besag-York-Mollié to capture spatial autocorrelation, to estimate the spatiotemporal distribution of ICC incidence among women diagnosed with HIV. We also examined the association of deprivation, access to health (using the number of health facilities per municipality) and urbanicity with ICC incidence. We corrected our estimates to account for ICC case underascertainment, missing data and data errors.

**Results:**

We included 17,821 ICC cases and demonstrated a decreasing trend in ICC incidence, from 306 to 312 in 2004 and from 160 to 191 in 2014 per 100,000 person-years across all municipalities and corrections. The spatial relative rate (RR) ranged from 0.27 to 4.43 in the model without any covariates. In the model adjusting for covariates, the most affluent municipalities had a RR of 3.18 (95% Credible Interval 1.82, 5.57) compared to the least affluent ones, and municipalities with better access to health care had a RR of 1.52 (1.03, 2.27) compared to municipalities with worse access to health.

**Conclusions:**

The results show an increased incidence of cervical cancer in affluent municipalities and in those with more health facilities. This is likely driven by better access to health care in more affluent areas. More efforts should be made to ensure equitable access to health services, including mitigating physical barriers, such as transportation to health centres and strengthening of screening programmes.

**Supplementary Information:**

The online version contains supplementary material available at 10.1186/s12942-021-00283-z.

## Background

HIV positive women have an up to five times higher risk of developing invasive cervical cancer (ICC) compared with HIV negative women [1]. Persistent infection with high-risk variants of the human papillomavirus (HPV) is a necessary cause for ICC [2]. In 2020, Southern Africa and Eastern Africa had the highest burden of cervical cancer worldwide with an incidence rate of over 40 and 36 cases per 100,000 population per year respectively [3, 4].

The two regions also have a high burden of HIV: 54% of people living with HIV worldwide live in Southern and Eastern Africa [5]. A study looking at the global ICC estimated determine that in Southern Africa, 53% of ICC cases were attributable to HIV [6].

In 2019, the World Health Organization called for the elimination of cervical cancer as a public health threat [7–9]. ICC can be prevented through HPV vaccination and early detection and treatment of pre-cancerous cervical lesions. When implemented effectively, these methods should result in decreased disease burden. However, globally there are disparities in ICC burden, even amongst HIV positive women. A global study demonstrated that HIV positive women in South Africa had a substantially higher incidence of ICC compared to the rest of the world, even after the introduction of antiretroviral therapy (ART) [10]. Poor access to screening, long waiting times for treatment of pre-cancerous cervical lesions as well as the socioeconomic inequities that exist in the country contribute to the high burden of ICC in South Africa [11, 12].

Spatiotemporal analysis of ICC incidence among HIV positive women can be used for disease surveillance over time and space, and to identify potential causes of the unequal distribution of ICC incidence. In this nationwide study in South Africa during 2004–2014, we examine the spatial, temporal and spatiotemporal incidence of ICC among women diagnosed with HIV. We perform three different corrections on the incidence rates to account for under-ascertainment, missing data and data errors. We assess whether urbanicity, deprivation and number of health facilities in a municipality (proxy for access to health services) explained any geographical or spatiotemporal patterns.

## Methods

### Study setting

In 2016, South Africa was divided into 9 provinces and 213 municipalities (Additional file 1: Figures S5-6). The area of the municipalities varies from 828.1 km^2^ to 44,231.3 km^2^. The population in 2016 was 55.7 million, varying from 8895 to 4,949,347 across the municipalities. The ethnic breakdown amongst South African women was 80.6% Black, 8.1% White, 8.9% Mixed race and 2.4% Asian [13].

### Study population

We retrieved data on women diagnosed with ICC with an HIV positive status in South Africa for the period 2004–2014 from the South African HIV cancer match (SAM) study [14]. The SAM study aims to estimate cancer incidence in people living with HIV. It uses probabilistic record linkage methods to build a national cohort of people living with HIV from HIV-related laboratory tests stored at the National Health Laboratory Service (NHLS) and ICC cancer cases recorded in the National Cancer Registry (NCR) [14]. The HIV data includes laboratory HIV diagnostic tests like enzyme-linked immunosorbent assay (ELISA), qualitative and quantitative polymerase chain reaction (PCR) as well as the Western Blot. In addition, the HIV data includes HIV laboratory monitoring tests such as CD4 cell counts and HIV RNA viral loads [14]. The NCR is a nationwide, pathology-based cancer registry in South Africa with compulsory cancer reporting of cancer cases by both private and public laboratories since 2011 [15]. The NHLS is the largest diagnostic pathology service in South Africa, providing health services to about 80% of the population of South Africa (almost entirely users of public health facilities) [15].

We included ICC cases diagnosed within 2 years prior to a first HIV laboratory test or later, assuming that women would have been HIV-positive on average for at least 2 years prior to the test. The NHLS collects addresses of facilities where HIV tests are performed. We geocoded these addresses using Google Maps and linked them with corresponding municipalities. We assumed that the municipality of residence is the municipality where the women received the HIV-related test result.

We estimated the number of person-years of women diagnosed with HIV in each municipality using the Thembisa model (version 4.3; https://www.thembisa.org/downloads) [16]. Thembisa is a mathematical model of the South African HIV epidemic, which provides provincial counts of cases diagnosed with HIV, stratified by age and sex. Since the Thembisa model does not provide estimates by municipality, we applied weights (by age, year and municipality) using NHLS data on women diagnosed with HIV to obtain municipal HIV estimates (see Additional file 1: Text S1.1 for the weight calculation and Figure S1 for the exclusion criteria of the NHLS).

### Outcome

The NCR classifies cancer diagnoses according to the International Classification of Diseases for Oncology third edition (ICD-O-3), excluding pre-cancerous lesions [17]. We included cervical cancers coded C53.0, C53.1, C53.8 and C53.9.

### Co-variates

We examined socioeconomic differentials across space using a multiple deprivation rank as determined in 2011 [18]. The most deprived ward is given a rank of 1. To adjust for differences in access to health care, we considered the urbanicity of the municipalities (urban–rural) as retrieved from the National Department of Health data dictionary which was last updated in 2019 (Additional file 1: Figure S2), and the number of health facilities per municipality 2004–2014 from NHLS, as a proxy for access to health services (Additional file 1: Figure S3) [19]. The health facility information in the NHLS dataset refers to primary and secondary care clinics and hospitals as well as regional and national hospitals from which the HIV test was requested. The spatial and spatiotemporal analysis was based on the addresses of these health facilities.

### Statistical methods

We used Bayesian hierarchical models to investigate the spatial and spatiotemporal distribution of ICC incidence among women diagnosed with HIV in South Africa. We first performed model selection without incorporating any covariates. Briefly, we used a re-parameterisation of the Besag-York-Mollié model to model the spatial autocorrelation [20–22]. This reparameterization includes a standard deviation hyperparameter that controls the variation of the field and a mixing hyperparameter indicating the proportion of the field that is dominated by strong spatial variation (ICAR prior [20]) as opposed to overdispersion. Random walk (RW) processes of order one were used to estimate the temporal random effect (on yearly resolution) and the random effects for the different age groups (15–19, 20–24, …, 75–79, > 80 years). We fitted a series of models taking all possible combinations of the aforementioned random effects and also considered a type I interaction (interaction between an unstructured temporal and spatial random effects) [23]. We used penalized complexity priors for all the hyperparametes (Additional file 1: Text S1.2) [21, 24]. To examine model performance and select the best performing model we derived the deviance information criterion (DIC), the Watanabe–Akaike information criterion (WAIC), and the mean logarithmic score (CPO) [25]. In a second step, we used the model that fitted best and adjusted for the selected covariates. The model of best fit was determined as the one that minimizes the DIC, WAIC and CPO. We categorised the deprivation rank and number of health facilities per municipality in deciles to allow flexible fits. Additional file 1: Text S1.2 provides further information about the model specification.

### Incidence corrections

We report four different estimates of the ICC incidence among women diagnosed with HIV. The first incidence estimates (‘no correction’) are from the best fitting model (without covariates).

The first correction (‘correction I’) accounts for the potential diagnosis of HIV in the private sector. NCR includes cases diagnosed in the private and public sector. The correction assumes that if an NCR case is diagnosed in the private sector, then the women are likely to have received the HIV diagnosis and care in the private sector, too. Out of the total 57,161 ICC cases diagnosed during 2004–2014 in South Africa, 32,564 were not linked with an HIV case. However, out of the 32,564, only 5,077 (16%) were diagnosed in the private sector. We assumed that (57,161–32,564)/57,161 = 43% of these cases are HIV related. Thus, for correction I, we oversampled 5077*0.43 = 2,183 cases (on average 1,596 remained after the inclusion criteria) from the linked ICC cases that were treated in the private sector. To account for the sampling uncertainty, we fitted the model 100 times and averaged over the samples using Bayesian model averaging [26].

For the second correction (‘correction II’), we used information about Kaposi sarcoma (KS). KS is a rare cancer in HIV-negative people but the most common cancer in people living with HIV [27]. In the SAM study, 70% of the 23,046 KS cases could be linked probabilistically to an HIV test. Out of the KS cases not linked, 1890 (8.2%) were diagnosed in the private sector. If we assume that all KS cases diagnosed from 2004 to 2014 occurred among people living with HIV, then the remaining 30%–8.2% = 21.8% missing HIV tests may be due to under-ascertainment of HIV, data errors or inaccuracies preventing linkage. To correct for the above, we calculated the proportion of unlinked KS cases by municipality excluding those treated in the private sector (because correction I accounts for that), and corrected the recorded numbers of ICC cases using the KS linked proportions (Additional file 1: Text S1.3). The third correction combines, by means of summing, the additional cases from corrections I and II (referred to as ‘full correction’).

We report median posterior and 95% credibility intervals (CrIs) for the temporal ICC incidence rate and performed age standardisation based on the world population weights [28]. We report median posterior of the exponential of the spatial random effects (spatial relative rates or spatial RRs; relative to the national average incidence rate over time), RRs for the effect of the selected covariates (relative to the baseline) and posterior probabilities (that the RRs is higher than 1). We used integrated nested Laplace approximation (INLA) for Bayesian inference [29]. The data and the code used for the analysis are online available https://github.com/gkonstantinoudis/CervixHIVRSA.

## Results

### Study setting and population

**Fig. 1:**
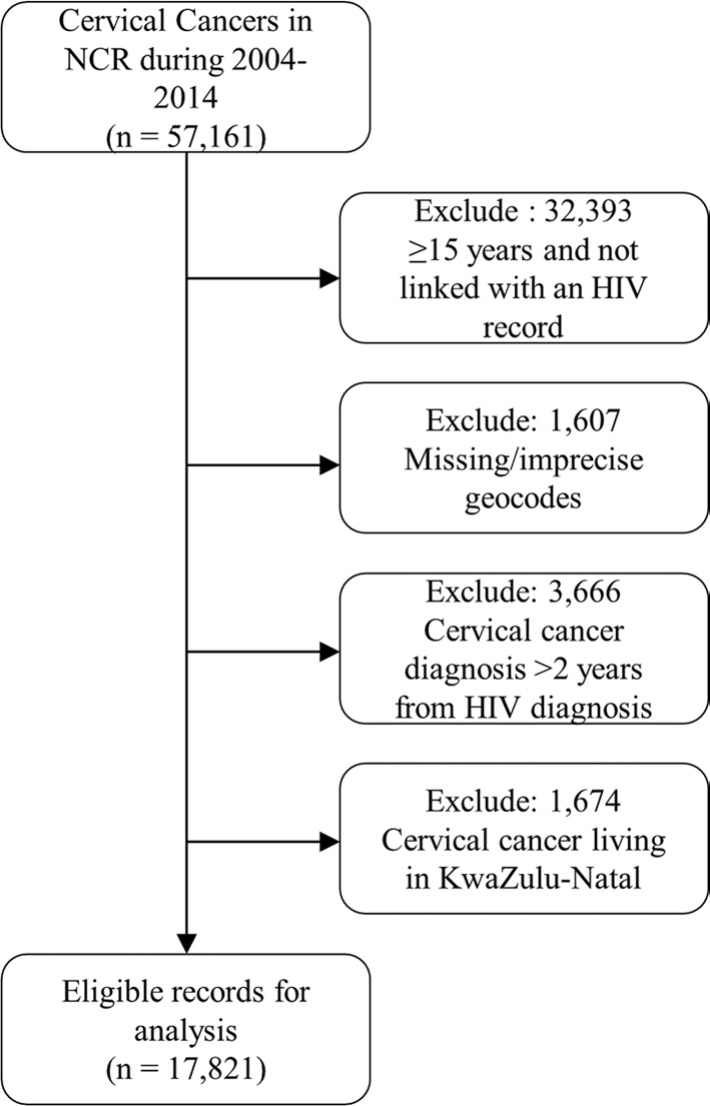
Flow of selection of eligible cervical cancer cases

We identified 57,161 ICC cases diagnosed from 2004 to 2014 in South Africa in the SAM study. We excluded 32,393 cases not linked with an HIV record and ≥ 15 years old, 1607 with missing or imprecise geocodes, 3666 with a ICC diagnosis 2 years or more before their first HIV related laboratory test and 1674 cases residing in KwaZulu-Natal leaving us with 17,821 (~ 32%) cases for the main analysis (Fig. 1). We excluded the KwaZulu-Natal province because it started contributing data to NHLS only in 2010. With correction I, the total number of cases varied from 19,380 to 19,466. It was 21,446 for correction II and varied from 23,005 to 23,091 for the full correction. The total person-years (PY) at risk among women diagnosed with HIV during 2004–2014 were 24,059,679.

### Model selection

Based on the DIC, WAIC and CPO the model with the best fit was the one with random effects for age, time, space and a space–time interaction (Additional file 1: Table S1).

### Co-variates

**Fig. 2:**
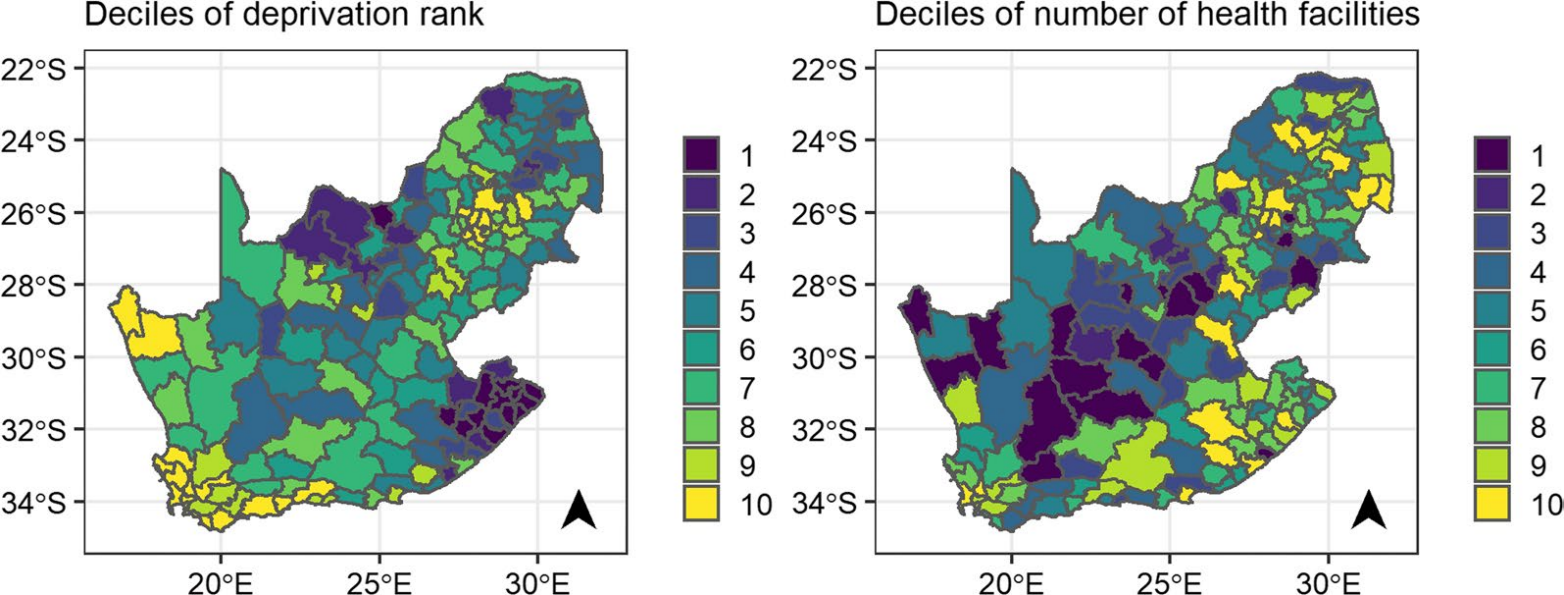
Maps of the deciles of the deprivation rank and number of health facilities per municipality

The spatial distribution of the deciles of deprivation rank and number of health facilities is shown in Fig. 2. The spatial distribution of urbanicity is shown in the Additional file 1: Figure S2. There were 23 urban and 190 rural municipalities in South Africa in 2016. Overall, we observe substantial spatial variation for all three covariates.

### Overall incidence

The median overall ICC incidence during 2004–2014 from the best fitting model was 294 (95% CrI: 174, 498) for no correction, 297 (95% CrI: 163, 573) for correction I, 310 (95% CrI: 170, 494) for correction II and 312 (95% CrI: 171, 510) for the full correction, per 100,000 PY, among women diagnosed with HIV (Additional file 1: Table S2).

### Temporal trends

**Fig. 3:**
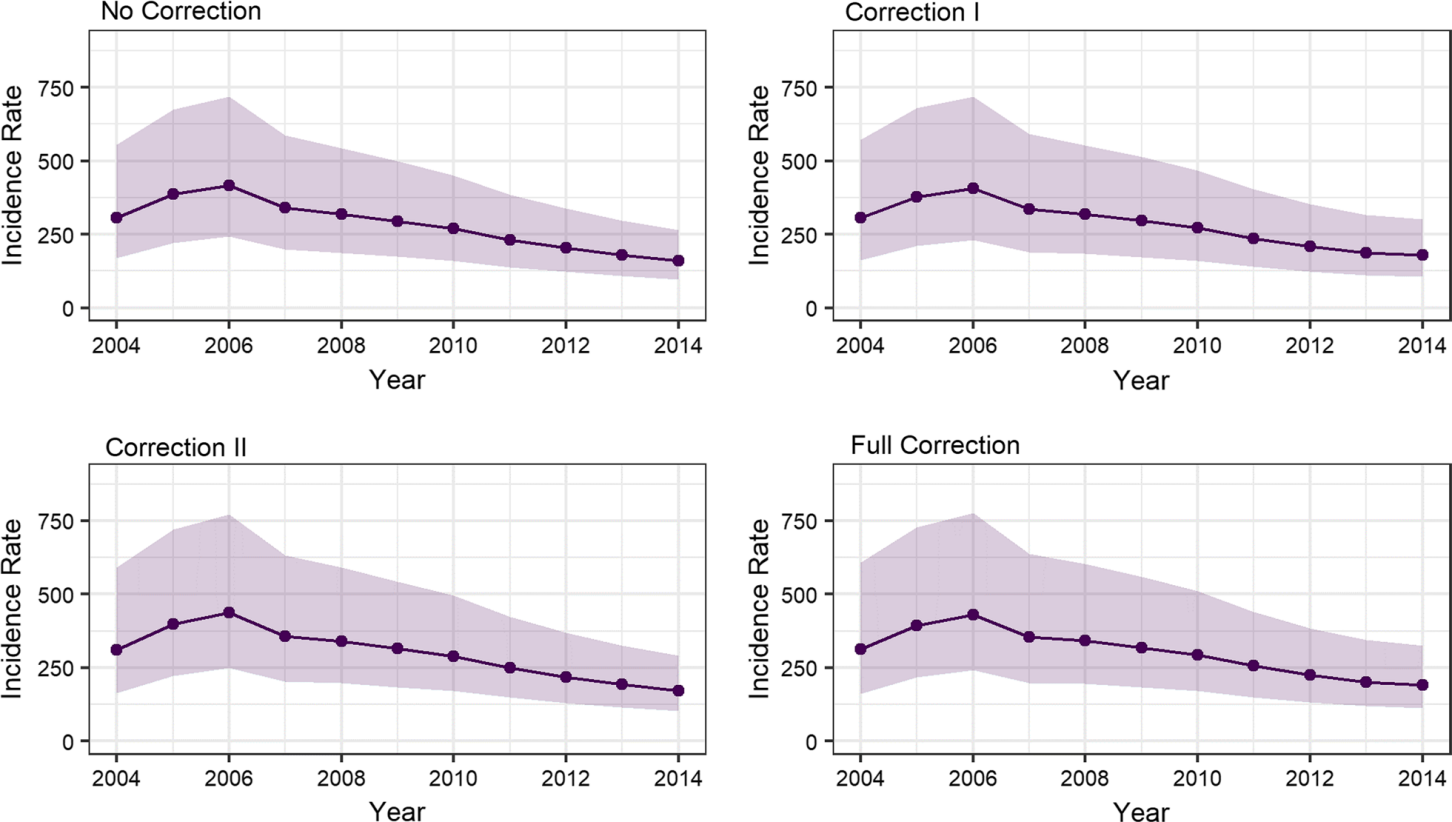
Yearly age-standardised incidence rate per 100,000 person years for women living with HIV in South Africa

Figure 3 shows that the annual median age-standardized ICC incidence rate per 100,000 PY of women diagnosed with HIV in South Africa decreased during 2004–2014. In 2004, the incidence rate varied from 306 (95% CrI: 169, 555) for no correction, to 306 (95% CrI: 163, 573) for correction I, and to 310 (95% CrI: 164, 589) for correction II and 312 (95% CrI: 160, 609) for the full correction, per 100,000 PY (Table S2 in Additional file 1:). In contrast, in 2014 the incidence rate varied from 160 (95% CrI: 96, 265) for no correction, to 179 (95% CrI: 106, 303) for correction I, to 172 (95% CrI: 103, 290) for correction II and to 191 (95% CrI: 113, 326) for the full correction, per 100,000 PY (in Additional file 1: Table S2).

### Spatial patterns

**Fig. 4:**
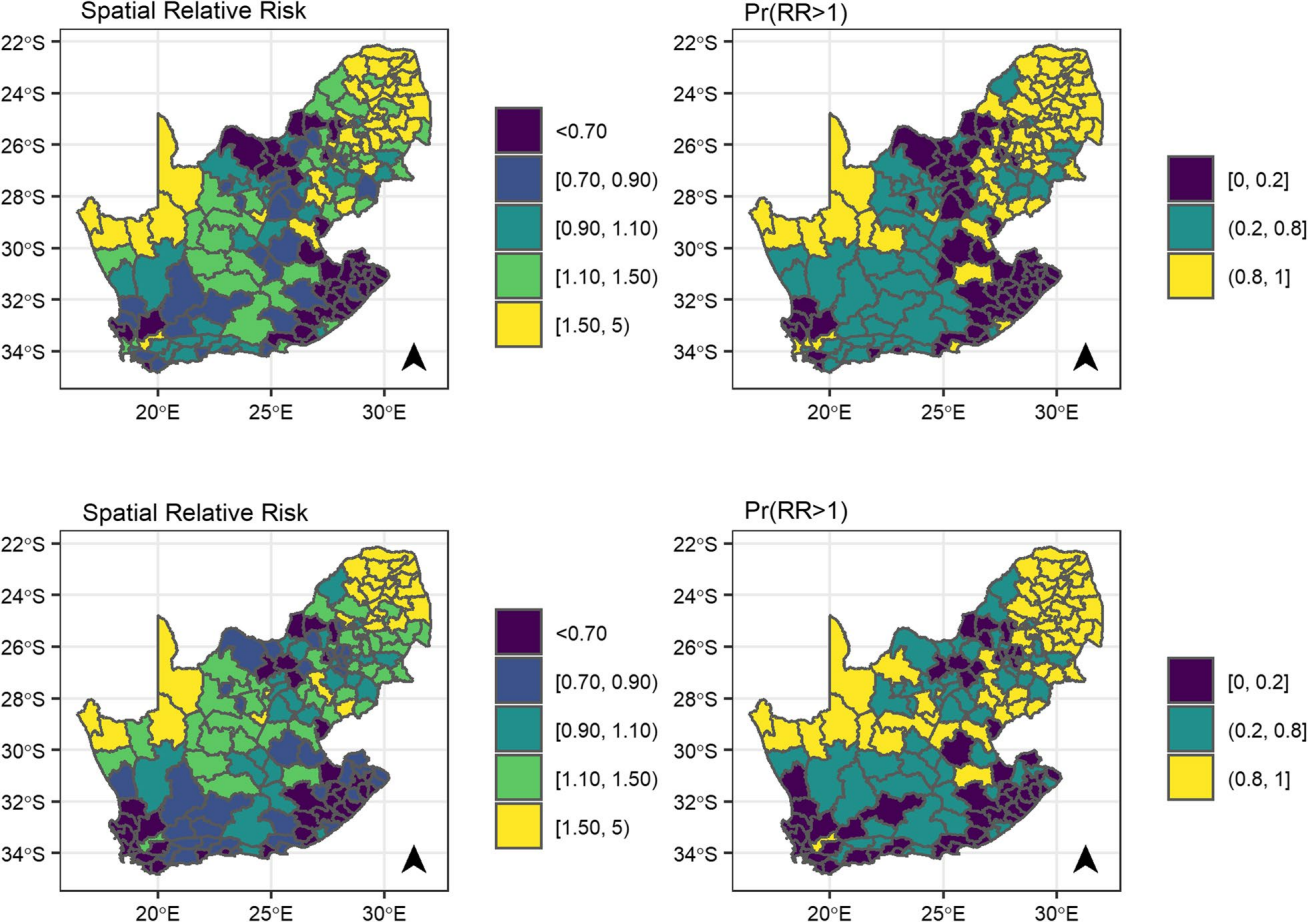
Median posterior of spatial relative risk (exponential of the spatial random effect) and posterior probability. The posterior probability that relative risk is larger than 1 of cervical cancer compared to the national average during 2004–2014 for the model without any correction and covariates (top panels) and the fully adjusted model without any correction (bottom panels)

Figure 4 shows the median posterior spatial RR and posterior probability of ICC across women diagnosed with HIV in the no correction model without covariates (top panels) and the no correction model adjusted for the selected covariates (bottom panels). For the model without covariates, the spatial RR ranged from 0.27 (95% CrI: 0.16, 0.42) to 4.43 (95% CrI: 3.71, 5.28). There was attenuation of associations when adjusting for covariates with the RR varying from 0.35 (95% CrI: 0.18, 0.64) to 4.10 (95% CrI: 2.90, 5.78). Nevertheless, the spatial variation remained strong after having adjusted for the selected covariates, with the mixing hyperparameter being 0.77 (0.45, 0.95), Additional file 1: Table S3. RR and posterior probabilities were larger in the province of the Northern Cape (north-western region of the map) and the Limpopo, Gauteng and Mpumalanga provinces (north-eastern part of the map) (Additional file 1: Figure S3). The results across the incidence corrections are similar, Additional file 1: Figure S7.

### Spatiotemporal patterns

**Fig. 5:**
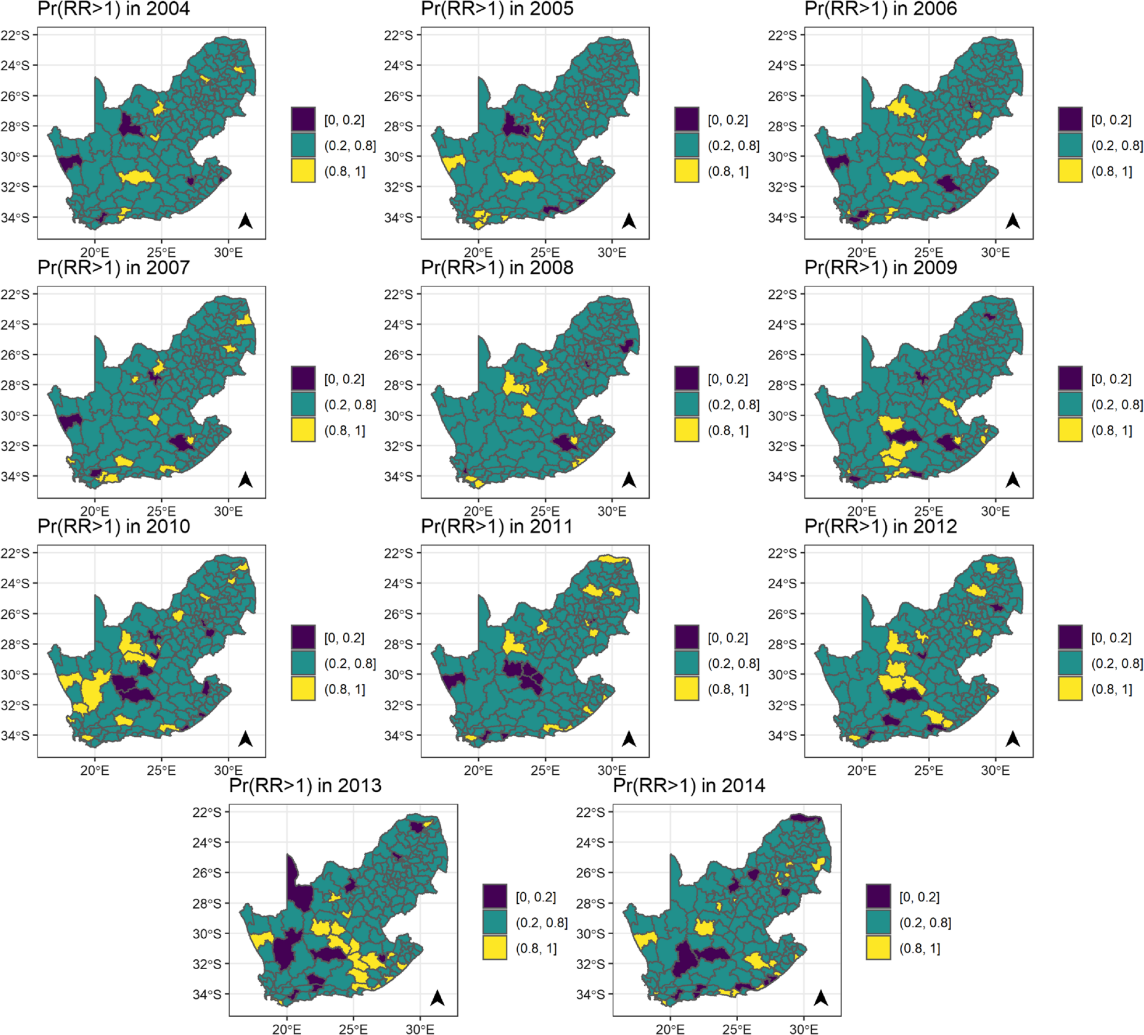
Posterior probability that the spatiotemporal relative risk (relative to the national average over time and space) of cervical cancers among women living with HIV in South Africa is higher than 1. This figure is based on the model without any covariates and corrections

The spatial differences have not attenuated over the years, and the patterns do not seem to be consistent in both ‘no correction’ models, with and without covariates (Fig. 5 and Additional file 1: Figure S8). The results across the incidence corrections are similar, Additional file 1: Figure S9–15.

### Influence of covariates

We assessed the influence of covariates in the no correction model (Table 1). The RR comparing the least with the most deprived municipalities was 3.56 (95% CrI: 2.16–5.89) in the univariable model, and 3.18 (95% CrI: 1.82–5.57) in the multivariable model. The RR in municipalities with the highest number of health facilities was 1.83 (95% CrI: 1.27–2.64) in the univariable model, and 1.52 (95% CrI: 1.03–2.27) in the multivariate model, compared to municipalities with the lowest number of health facilities. There was little evidence of a difference in ICC incidence across urban and rural municipalities, both in the univariable (1.19, 95% CrI 0.94–1.50) and multivariable model (0.85, 95% CrI 0.65–1.12).

**Table 1:** Median posterior relative rate and 95% credibility intervals of the effect of the covariates

		Univariable models			Mutlivariable model		
Co-variates		Median	95% CrI	Pr(RR > 1)	Median	95% CrI	Pr(RR > 1)
Urbanicity	Rural	1.00	–	1.00	1.00	–	1.00
	Urban	1.19	(0.94, 1.50)	0.92	0.85	(0.65, 1.12)	0.12
Deprivation rank	1st Decile	1.00	–	1.00	1.00	–	1.00
	2nd Decile	1.81	(1.16, 2.82)	1.00	1.77	(1.13, 2.78)	0.99
	3rd Decile	1.84	(1.19, 2.86)	1.00	1.68	(1.07, 2.66)	0.99
	4th Decile	2.14	(1.35, 3.40)	1.00	1.97	(1.23, 3.16)	1.00
	5th Decile	1.76	(1.09, 2.83)	0.99	1.83	(1.10, 3.04)	0.99
	6th Decile	2.22	(1.41, 3.49)	1.00	1.97	(1.23, 3.17)	1.00
	7th Decile	2.40	(1.50, 3.85)	1.00	2.33	(1.43, 3.79)	1.00
	8th Decile	2.82	(1.78, 4.50)	1.00	2.69	(1.66, 4.35)	1.00
	9th Decile	3.21	(1.98, 5.21)	1.00	2.88	(1.75, 4.74)	1.00
	10th Decile	3.56	(2.16, 5.89)	1.00	3.18	(1.82, 5.57)	1.00
Number of health facilities	1st Decile	1.00	–	1.00	1.00	–	1.00
	2nd Decile	1.16	(0.68, 1.98)	0.71	1.14	(0.67, 1.92)	0.69
	3rd Decile	0.97	(0.66, 1.42)	0.43	0.90	(0.62, 1.31)	0.29
	4th Decile	1.14	(0.77, 1.68)	0.74	1.04	(0.71, 1.55)	0.59
	5th Decile	1.24	(0.86, 1.81)	0.87	1.06	(0.73, 1.54)	0.61
	6th Decile	1.17	(0.79, 1.74)	0.78	1.07	(0.72, 1.60)	0.64
	7th Decile	1.15	(0.80, 1.67)	0.77	1.05	(0.72, 1.56)	0.61
	8th Decile	1.31	(0.91, 1.89)	0.93	1.18	(0.81, 1.71)	0.81
	9th Decile	1.35	(0.93, 1.97)	0.95	1.24	(0.85, 1.83)	0.87
	10th Decile	1.83	(1.27, 2.64)	1.00	1.52	(1.03, 2.27)	0.98

CrI, Credibility intervals; Pr(RR > 1), Posterior probability that the relative rate is larger than 1. The models were adjusted for urbanicity, deprivation rank and number of health facilities as a proxy for access to health

## Discussion

### Main findings

In this study, we determined the spatial, temporal and spatiotemporal variation of ICC incidence in women diagnosed with HIV in South Africa, and assessed whether urbanicity deprivation and number of health facilities explained any geographical or spatiotemporal patterns. We observed an overall decreasing trend in ICC incidence in women diagnosed with HIV in South Africa during 2004–2014. We found a higher relative incidence in the Northern Cape and in the Limpopo, Gauteng and Mpumalanga provinces. Regarding the spatiotemporal patterns, the spatial differences did not decrease over time. Also, HIV positive women residing in the least deprived municipalities or in municipalities with larger numbers of health facilities were more likely to get diagnosed with ICC.

### Discussion in the context of previous studies

The incidence observed across all corrections is consistent with other studies on ICC incidence in women living with HIV in South Africa. Two hospital-based cohort studies determined crude incidence rates that ranged from 447 to 506 ICC cases/100,000 person-years [10, 30]. These results reflect the high ICC burden in HIV positive women in South Africa compared to the overall crude incidence rate of ICC in the general South African population. The latter experienced an incidence of 22.68 ICC cases/100,000 of the population as of 2016 [31]. Other HIV and cancer registry linkage studies in Africa have shown variable crude incidence rates in women living with HIV. In Nigeria, investigators observed an incidence of 7.8/ 100,000 person-years [32] whilst in Uganda, the incidence reported was 70/ 100 000 person-years, with the incidence being highest in the 35–44 age group at 200/100,000 person-years [33]. These differences in ICC incidence might potentially reflect differences in access to ICC screening and diagnosis in the different countries.

Although CC incidence in women diagnosed with HIV is still high compared to the general population, we observed a decreasing trend in ICC incidence after an initial increase between 2004 and 2006, similar to the decline that was observed in high income countries after the introduction of ART [34, 35]. However, in other African settings, the ICC incidence trends either remained unchanged or increased even after the introduction of ART [33, 36]. The decline in incidence in our study might reflect the HIV testing patterns in South Africa across the study period. Initially, fewer people were tested for HIV [37] and those tested had an increased burden of disease and lower CD4 cell counts [38]. Increased ICC incidence has been associated with very low CD4 cell counts compared to higher CD4 cell counts. With time, as HIV testing expanded, more women had access to timely HIV diagnosis and treatment before advanced disease, thus reducing the likelihood of ICC. The timely access might have resulted in the subsequent decline in ICC incidence from 2006 amongst women diagnosed with HIV. ART reduces the prevalence of high-risk HPV infections and cervical pre-cancerous lesions. Still, the effect is reduced in individuals initiating ART at an advanced HIV stage and in women with advanced pre-cancerous lesions [39–41]. The expansion of the national ART programme, which started in 2004, ensured that more people had access to HIV treatment and subsequently improved CD4 cell counts. This expansion could also explain the subsequent decline in incidence from 2006 to 2014 as women had a lower burden of disease and better access to ART. However, in some studies this has not been supported. In Botswana, no significant change in ICC incidence was observed after ART expansion [36]. Spatial analyses have been used in ICC studies to determine disparities in incidence and mortality as well as the factors associated with the unequal distribution of ICC burden [30, 42–45]. However, all these studies focused on the general population regardless of HIV status. Previous studies examining the spatial trends in the USA, Thailand and Cuba have reported variations in ICC incidence driven by a variety of factors [42, 46, 47]. In Thailand, Zhao et al. observed differences in ICC incidence in districts in the Songhkla province driven by sexual behaviour and migration patterns [47]. A similar study in Cuba showed that differences in screening programme coverage did not account for the spatial differences in ICC incidence, which were better explained by differences in lifestyle and socioeconomic status [43]. In the USA, the disparities were attributed to ethnicity, socioeconomic status and screening coverage [42]. Low socioeconomic status of areas and low screening rates were associated with an increased ICC incidence. Another spatiotemporal study in Texas, USA demonstrated that poor education, African and Hispanic ethnicities and low socioeconomic status was associated with a high ICC incidence [48]. In South Africa, Black women have a higher incidence of ICC compared to other races. The incidence of ICC amongst black women in 2017 was 28.25 per 100 000 women compared to 8.29, 13.66 and 15.24 in Asian, Mixed race and White women respectively [31]. This is largely attributed to differences in HIV prevalence as well as unequal access to health care services. However, in our study data on ethnicity was not available.

In our study, more affluent areas had a higher incidence of ICC. People living in the least deprived municipalities were about three times more likely to be diagnosed with ICC. This points towards disparities in ICC, driven by unequal access to services due to socioeconomic circumstances. In Uganda, a spatial analysis of ICC in women, regardless of HIV status, showed that areas of low socioeconomic status had decreased access to care, including ICC screening [44]. This can lead to a spurious negative association between ICC incidence and socioeconomic status, as areas of low socioeconomic status would appear to have a lower burden of disease when the ICC cases are not being diagnosed. Some districts in South Africa have pronounced shortages of health care staff, with multiple vacant posts and ill-equipped facilities [49], which affects access to care. In addition, utilisation of public health sector facilities in South Africa has been shown to be higher among people from least deprived areas, with individuals from deprived areas having limited access to public health sector services [50].

We observed a relationship between the number of facilities per municipality and ICC incidence in women diagnosed with HIV: the greater the number of health facilities, the higher the ICC incidence. In our study, we used the number of health facilities per municipality as a proxy for differences in access to health care services. This points to the effect of inequalities in access to care on ICC incidence. In the United States, in urban areas, a larger distance to the health facility was a barrier to accessing cervical cancer care and treatment [51]. However, the same was not observed in rural areas, where long distances were associated with better access, with the suggested reason being the familiarity and tolerance to long-distance travel for health services in rural areas [51]. A qualitative study in Botswana determined thematic issues that affected access to ICC treatment and care. Among those were contextual issues like distance from facility, other commitments and transportation issues as well as health system issues such as long waiting times for appointments with health care workers and for test results [52]. In South Africa, it is estimated that for every additional health care professional per year, the likelihood of cervical cancer screening improved by 1% [53].

Deprivation, access to health and urbanicity did not explain all of the observed spatial variation. The remaining spatial trends can point towards geographical health inequalities, potentially generated by different screening programmes and municipality level health policies. Differences in HPV prevalence might also influence the ICC incidence patterns. There was no readily available nationwide screening coverage data or ethnicity data at the municipality level for women diagnosed with HIV at the time of analysis.

## Strengths and limitations

To our knowledge, our study is the first nationwide study to describe the temporal, spatial and spatiotemporal variation in ICC incidence by municipality amongst women diagnosed with HIV in South Africa. Given that our data sources cover the majority of the South African population, and given the incidence adjustments we made, our incidence estimates are expected to cover the majority of women diagnosed with HIV in South Africa. The correction factors reduced the selection bias introduced through our data sources and the record linkage process. However, our study also has several limitations. In our study, the population under observation was specific to a sub-population of women who knew their HIV status and thus had contact with health care facilities. This contact increases the probability of being referred for ICC screening and treatment when compared to women who are unaware of their HIV status. As a result, the results cannot be generalised to women who have not been diagnosed with HIV. According to a survey by the Human Science Research Council, 47.5% of women aged 15 years and older were unaware of their HIV positive status in 2017 [54]. Although we introduced several incidence corrections, women without health care access are missing from our study. The ecological nature of our analysis did not allow us to adjust for CD4 counts that might influence ICC incidence. We could not adjust for screening coverage due to the absence of nationwide screening data for women living with HIV. While disparities in ICC incidence by ethnicity have been shown in South Africa, we lacked information on ethnicity in our data which might have explained some of the differences in ICC across municipalities [31]. Prior to 2011, NCR consisted mainly of cancer cases reported by public laboratories. In 2011, compulsory cancer reporting was established by both private and public laboratories [15, 55]. This is not expected to have substantially affected ICC reporting in HIV-positive women, since almost all HIV-positive women receive treatment through public sector services. Lastly, in our study, we could not assess any of the aforenmentioned different metrics pertaining to access. We should also note that we assumed that the municipality of the health facility is the same as the municipality of residence. This assumption may be problematic as people can move to other provinces and municipalities for healthcare. For instance, municipalities with more facilities might have better accessibility and shorter waiting times and hence people might choose to go there instead. Thus, we cannot rule out that the observed effect can be driven by this assumption.

## Conclusion

In conclusion, although the burden of ICC among women diagnosed with HIV has decreased over time, there was strong spatial variation. Individuals from less deprived areas and areas with more health facilities are more likely to be diagnosed with ICC compared to those from most deprived areas or areas with fewer health facilities. The geographical discrepancies persisted after adjusting for the aforementioned factors, which could reflect spatial differences in screening policies across women living with HIV. More efforts should be made to ensure equitable access to health services, including mitigating physical barriers, such as transportation to health centres, strengthening of screening programmes, and nationwide HPV vaccination implementation in populations living with HIV. Special attention should be paid to women living in low socioeconomic areas.

## Supplementary Information

**Additional file 1: Table S1. **Deviance information criterion (DIC), Watanabe-Akaike information criterion (WAIC) and mean logarithmic score (CPO) for the different models considered. For the notation. **Table S2.** Annual median and 95% Credibility intervals (CrI) for the incidence rate of cervical ancers among HIV positive women per 100,000 person years in South Africa for the different corrections considered. **Table S3.**Results of the model with spatial, temporal and spatiotemporal interaction and depri-vation and urbanicity for the different Thembisa denominators. **Figure S1. **Flowchart for the exclusion criteria used to calculate weights using data from the Na-tional Health Laboratory Service (NHLS) to disaggregate the Thembisa provincial es-timates. **Figure S2. **The spatial variation of urbanicity (urban/rural) in South Africa. **Figure S3. **The number of health facilities by municipality and year (left panel) and by municipality in 2014 (right panel). **Figure S4. **The spatial variation of socioeconomic index in South Africa. A rank of 1 denotes the most deprived area. **Figure S5. **Provinces in South Africa in 2016 [ROSEA]. **Figure S6. **Municipalities in South Africa in 2016 [ROSEA]. **Figure S7. **Median posterior of spatial relative risk (exponential of the spatial random effect) and posterior probability using correction I, II and full correction. **Figure S8. **Posterior probability that the spatiotemporal relative risk (relative to the national av-erage over time) is higher than 1 of cervical cancers among women living with HIV in South Africa, using the no correction model adjusted for the selected covariates.

## Data Availability

The aggregated data will be made available at https://github.com/gkonstantinoudis/CervixHIVRSA.

## Abbreviations

ART: Antiretroviral therapy
CPO: Mean logarithmic score
CrI: Credibility Intervals
DIC: Deviance Information Criterion
ELISA: Enzyme-linked immunosorbent assay
HPV: Human Papillomavirus
ICAR: Intrinsic conditional autoregressive process
ICC: Invasive cervical cancer
ICD-O-3: International Classification of Diseases for Oncology third edition
INLA: Integrated Nested Laplace approximation
KS: Kaposi sarcoma
NCR: National Cancer Registry
NHLS: National Health Laboratory Service
PCR: Polymerase Chain Reaction
PY: Person Years
RW: Random Walk
RR: Relative rates
SAM: South African HIV cancer match
WAIC: Watanabe-Akaike Information Criterion
