# Supplementary material for: Spatiotemporal modelling and mapping of cervical cancer incidence among HIV positive women in South Africa: a nationwide study
Source: Int J Health Geogr. 2021 Jun 29;20:30. doi: 10.1186/s12942-021-00283-z (PMC8244168; doi:10.1186/s12942-021-00283-z)
Supplement: Supplementary file 1 — Additional file 1: Table S1. Deviance information criterion (DIC), Watanabe-Akaike information criterion (WAIC) and mean logarithmic score (CPO) for the different models considered. For the notation. Table S2. Annual median and 95% Credibility intervals (CrI) for the incidence rate of cervical ancers among HIV positive women per 100,000 person years in South Africa for the different corrections considered. Table S3.Results of the model with spatial, temporal and spatiotemporal interaction and depri-vation and urbanicity for the different Thembisa denominators. Figure S1. Flowchart for the exclusion criteria used to calculate weights using data from the Na-tional Health Laboratory Service (NHLS) to disaggregate the Thembisa provincial es-timates. Figure S2. The spatial variation of urbanicity (urban/rural) in South Africa. Figure S3. The number of health facilities by municipality and year (left panel) and by municipality in 2014 (right panel). Figure S4. The spatial variation of socioeconomic index in South Africa. A rank of 1 denotes the most deprived area. Figure S5. Provinces in South Africa in 2016 [ROSEA]. Figure S6. Municipalities in South Africa in 2016 [ROSEA]. Figure S7. Median posterior of spatial relative risk (exponential of the spatial random effect) and posterior probability using correction I, II and full correction. Figure S8. Posterior probability that the spatiotemporal relative risk (relative to the national av-erage over time) is higher than 1 of cervical cancers among women living with HIV in South Africa, using the no correction model adjusted for the selected covariates. [file 12942_2021_283_MOESM1_ESM.pdf]

Online Supplement: Spatiotemporal mapping of cervical cancer incidence among women living with HIV in South Africa: A nationwide study

Tafadzwa Dhokotera[1], Julien Riou[1], Lina Bartels[1], Eliane Rohner[1], Frederique Chammartin[1], Elvira Sign[2,4], Victor Olago[2,4], Mazvita Sengavi[2,4], Matthias Egger[1], Julia Bohlius[1], and Garyfallos Konstantinoudis*[1,5]

[1] *Institute of Social and Preventive Medicine (ISPM), University of Bern, Bern*

[2] *Centre for Infectious Disease Epidemiology and Research, University of Cape Town, South Africa*

[3] *Centre for Infectious Disease Epidemiology and Research, School of Public Health and Family Medicine, University of Cape Town, South Africa*

[4] *Division of Epidemiology and Biostatistics, School of Public Health, University of the Witwatersrand, Johannesburg, South Africa*

[5] *Epidemiology and Biostatistics department, School of Public Health, Imperial College London, United Kingdom*

*Corresponding author; e-mail:g.konstantinoudis@imperial.ac.uk

Contents

S1 Text **4**

S1.1 Thembisa model disaggregation 4

S1.2 Model description 6

S1.3 Correction II 7

List of Tables

S1 Deviance information criterion (DIC), Watanabe-Akaike information criterion (WAIC) and mean logarithmic score (CPO) for the different models considered. For the notation refer to Text S1.2. 9

S2 Annual median and 95% Credibility intervals (CrI) for the incidence rate of cervical cancers among HIV positive women per 100,000 person years in South Africa for the different corrections considered. 26

S3 Results of the model with spatial, temporal and spatiotemporal interaction and deprivation and urbanicity for the different Thembisa denominators. 27

List of Figures

S1 Flowchart for the exclusion criteria used to calculate weights using data from the National Health Laboratory Service (NHLS) to disaggregate the Thembisa provincial estimates. 10

S2 The spatial variation of urbanicity (urban/rural) in South Africa. 11

S3 The number of health facilities by municipality and year (left panel) and by municipality in 2014 (right panel). 12

S4 The spatial variation of socioeconomic index in South Africa. A rank of 1 denotes the most deprived area. 13

S5 Provinces in South Africa in 2016 [ROSEA]. 14

S6 Municipalities in South Africa in 2016 [ROSEA]. 15

S7 Median posterior of spatial relative risk (exponential of the spatial random effect) and posterior probability using correction I, II and full correction. 16

S8 Posterior probability that the spatiotemporal relative risk (relative to the national average over time) is higher than 1 of cervical cancers among women living with HIV in South Africa, using the no correction model adjusted for the selected covariates. . . . 17

S9 Median posterior of spatial relative risk (exponential of the spatial random effect) of cervical cancers compared to the national average during 2004-2014 for the model without any covariates. . . . 18
S10 Median posterior of spatial relative risk (exponential of the spatial random effect) of cervical cancers compared to the national average during 2004-2014 for the model without any covariates and using correction I. . . . 19
S11 Median posterior of spatial relative risk (exponential of the spatial random effect) of cervical cancers compared to the national average during 2004-2014 for the model without any covariates and using correction II. . . . 20
S12 Median posterior of spatial relative risk (exponential of the spatial random effect) of cervical cancers compared to the national average during 2004-2014 for the model without any covariates and using the full correction. . . . 21
S13 Posterior probability that the spatiotemporal relative risk (relative to the national average over time) is higher than 1 of cervical cancers among women living with HIV in South Africa, using correction I and the model without any covariates. . . . 22
S14 Posterior probability that the spatiotemporal relative risk (relative to the national average over time) is higher than 1 of cervical cancers among women living with HIV in South Africa, using correction II and the model without any covariates. . . . 23
S15 Posterior probability that the spatiotemporal relative risk (relative to the national average over time) is higher than 1 of cervical cancers among women living with HIV in South Africa, using full correction and the model without any covariates. . . . 24

S1 Text

S1.1 Thembisa model disaggregation

The Thembisa model (among other information) provides estimates of HIV prevalent cases and people diagnosed with HIV per province, year and age during 2004 and 2014. For our analysis we used people diagnoses with HIV as the denominator. To disaggregate the provincial HIV counts to the municipality unit we calculated weights based on information provided by the National Health Laboratory Service (NHLS). We focused on individuals with ≥ 2 tests, and we selected as date of HIV diagnosis the date

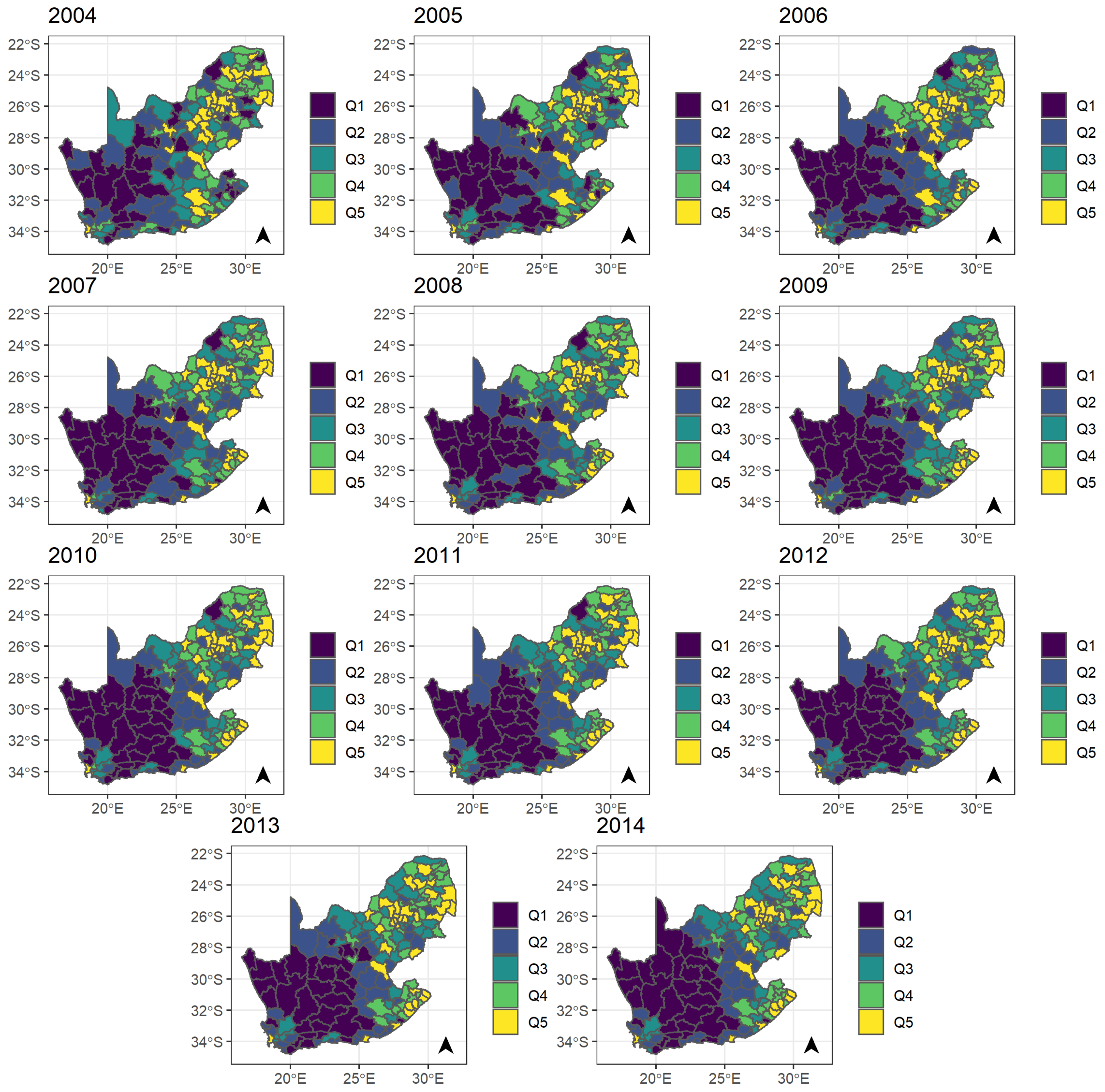

Figure 1. The quintiles of the population density per municipality of women diagnosed with HIV as resulted from disaggregating the Thembisa model using weights calculated from the National Health Laboratory Service dataset.

of the first recorded test/laboratory result. We assumed that the municipality where the first test was performed is the same as the municipality of residence of the HIV case.

Let P_{ijt} be the number of women diagnosed with HIV (based on NHLS) in the i–th municipality where $i = 1, \dots, n$, j–th province, with $j = 1, \dots, J$ and at year t, $t = 1, \dots, T$. We calculated province-specific municipality weights as $w_{ijt} = P_{ijt}/P_{\cdot jt}$ where $P_{\cdot jt} = \sum_{i \sim j} P_{ijt}$ where $i \sim j$ denotes the municipalities that belong to the j–th province at year t. To dissagregate the Thembisa model provincial counts $\tilde{P}_{\cdot jt}$ at year t, we multiplied them with the weights w_{ijt}, i.e. $\tilde{P}_{it} = w_{ijt} \cdot \tilde{P}_{\cdot jt}$, where $\tilde{P}_{it}$ is the Thembisa model municipality counts at year t.

To get the age dimension, we assumed that w_{ijt} is constant over the different k age groups considered (0-4, 5-9, ..., >80) and retrieved $\tilde{P}_{itk}$ (the Thembisa counts in the i–th municipality, t–th year and k–th age group). The output of the procedure is given on Figure 1 for all ages.

S1.2 Model description

Let A be an observation window divided in spatial units $A_1, A_2 \dots A_n$ (municipalities in South Africa). Let Y_{itk} be the counts of cervical cancer cases in the i–th municipality, t–th year and k–th age group. A general model formulation would be:

$$
\begin{aligned}
Y_{itk}|\lambda_{itk},\tilde{P}_{itk} &\sim \text{Poisson}(\lambda_{itk}\tilde{P}_{itk}) \\
\log(\lambda_{itk}) &= \beta_0 + \eta_k + \phi_i + w_t + \delta_{it} \\
\beta_0 &\sim \mathcal{N}(0, \infty) \\
\eta_k &\sim \text{RW1}(\sigma_1^2) \\
\phi_i &\sim \text{BYM2}(\boldsymbol{W}, \sigma_2^2, \rho) \\
w_t &\sim \text{RW1}(\sigma_3^2) \\
\delta_{it} &\sim \mathcal{N}(0, \sigma_4^2) \\
\sigma_1^2, \sigma_2^2, \sigma_3^2, \sigma_4^2, \rho &\sim \text{PCpriors}
\end{aligned}
$$

where λ_{itk} is the incidence rate and $\tilde{P}_{itk}$ is the population counts (as defined in Text S1.1) in the i–th municipality, t–th year and k–th age group. β_0 is an intercept term, η the age group random effect defined as a random walk of order 1 (RW1), ϕ the spatial random effect [Besag et al., 1991, Simpson et al., 2017], w_t a temporally structured random effect (RW1), δ the space-time interaction (we considered type I) of the spatial and temporal components. The type I interaction refers to unstructured overdispersion in time and space [Knorr-Held, 2000]. The hyperparameters $\sigma_1^2, \sigma_2^2, \sigma_3^2$, σ_4^2 are variances, ρ is the mixing parameter of the spatial field ϕ_i and $\mathbf{W}$ the neighborhood matrix. We used the queen contiguity weights for $\mathbf{W}$.

The priors for all the variance hyperparameters where set based on $\Pr(\sigma_i < 1) = 0.01$, for $i = 1, 2, 3, 4$ reflecting that is unlikely to have a risk $\exp(1) \approx 2.72$ times higher than the temporal, age specific, spatial average or spatiotemporal average. The mixing parameter ρ of the spatial field was selected based on $\Pr(\rho > 1) = 0.50$, reflecting our lack of knowledge whether the unstructured or spatially structured random effect should dominate the field. For more information about the PCpriors see Simpson et al. [2017].

S1.3 Correction II

Let $i = 1, \ldots, n$ be the number of municipalities, $j = 1, \ldots, J$ the provinces in South Africa and $t = 1, \ldots, T$ for the years. Let C_{ijtl} stand for the number of Kaposi sarcoma (KS) cases residing in the i–municipality, with their lab report sent to the j–th province in the t–th year and $l = 1$ to denote the linked cases (with the NHLS after performing the linkage) and 0 the unlinked.

For $l = 1$, the cases are linked with the NHLS thus the municipality of residence is known, and thus C_{it1} (the number of Kaposi sarcoma cases in the i–municipality, t–th year that are linked with the NHLS) is known. However, for the unlinked cases we only have the province where the cancer test was sent. Thus we do not have the C_{it0} (the number of Kaposi sarcoma cases in the i–municipality, t–th year that are not linked with the NHLS), but we have C_{jt0} (the number of Kaposi sarcoma cases in the j–th province, t–th year that are not linked with the NHLS). To calculate C_{it0} (the number of Kaposi sarcoma cases in the i–municipality, t–th year that are not linked with the NHLS), we calculated weights defined as: $w_{ijt} = C_{ijt1}/C_{jt1}$. The interpretation of these weights is: the proportion of tests (for cancer diagnosis) of the i–th municipality that are sent to the j–th province to be examined (at year t among the linked cancer cases). To approximate C_{it0}, we define $\tilde{C}_{it0} \approx \sum_j w_{ijt} = C_{jt0}$. Thus the correction factor is:

$$b_{it} = \frac{C_{it1}}{C_{it1} + \tilde{C}_{it0}}.$$

b_{it} takes values from 0 to 1, where 0 means that nothing is linked, whereas 1 that all cancer cases are linked.

We additionally excluded the KS cases not linked and treated in the private sector. Let K_{it0} be the KS cases not linked with NHLS and treated in the private sector, we can then write:

$$b_{it} = \frac{C_{it1} + K_{it0}}{C_{it1} + \tilde{C}_{it0}}$$

The output of the above procedure is shown in Figure 2. We note, that we aggregate in time to avoid having a lot of zeros in the data, making it hard to apply the correction on the model-based incidence output.

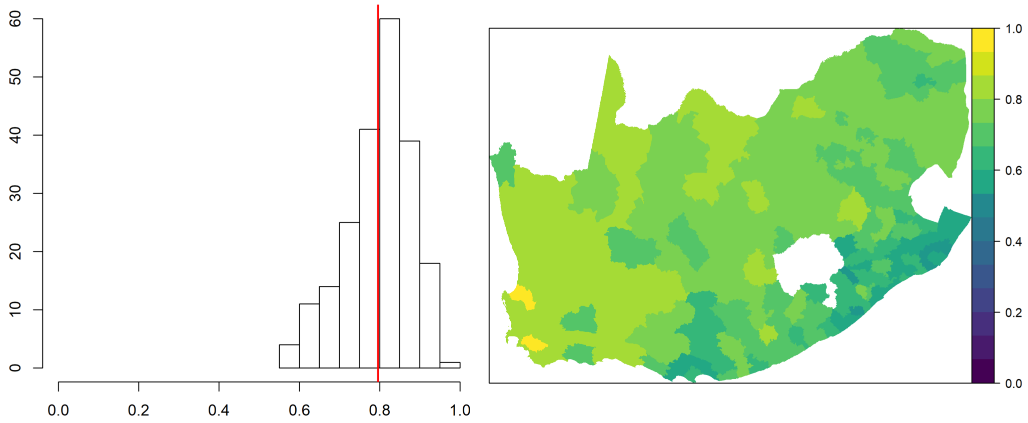

Figure 2. The correction II factor in space (right panel) and its histogram (left panel). The red line is the mean of the proportions.

β_0 is an intercept term, η_k the age effect, w_t the temporal effect, ϕ_i the spatial effect and δ_{it} the spatiotemporal effect.

Table S1: Deviance information criterion (DIC), Watanabe-Akaike information criterion (WAIC) and mean logarithmic score (CPO) for the different models considered. For the notation refer to Text S1.2.

models	DIC	WAIC	CPO
β_0	56694.15	56700.84	1.09
$\beta_0 + \eta_k$	35668.98	35685.81	0.69
$\beta_0 + w_t$	56419.44	56480.29	1.08
$\beta_0 + \phi_i$	52535.31	52699.83	1.01
$\beta_0 + \eta_k + w_t$	34110.17	34133.37	0.66
$\beta_0 + \eta_k + \phi_i$	31559.09	31606.54	0.61
$\beta_0 + w_t + \phi_i$	52276.15	52495.72	1.01
$\beta_0 + \eta_k + w_t + \phi_i$	30061.23	30093.06	0.58
$\beta_0 + w_t + \phi_i + \delta_{it}$	51853.77	53005.64	1.02
$\beta_0 + \eta_k + w_t + \phi_i + \delta_{it}$	29625.55	29726.33	0.57

CrI: Credibility intervals

$1/\sigma_1^2$ is the precision of the random walk of order 1 (RW1) of the age effect, $1/\sigma_2^2$ of the spatial field, $1/\sigma_3^2$ of the temporal effect, and $1/\sigma_4^2$ of the spatiotemporal interaction.

* The hyperparameters refer to the distribution of the logged random effects.

Figure S1: Flowchart for the exclusion criteria used to calculate weights using data from the National Health Laboratory Service (NHLS) to disaggregate the Thembisa provincial estimates.

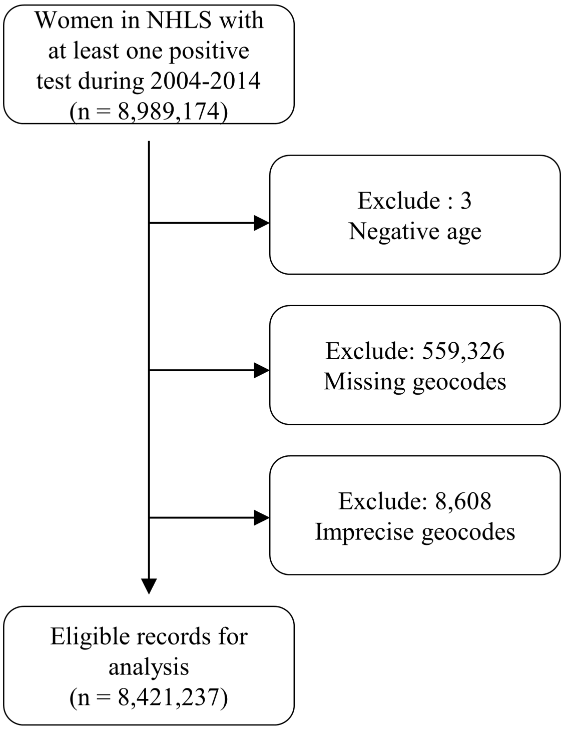

Figure S2: The spatial variation of urbanicity (urban/rural) in South Africa.

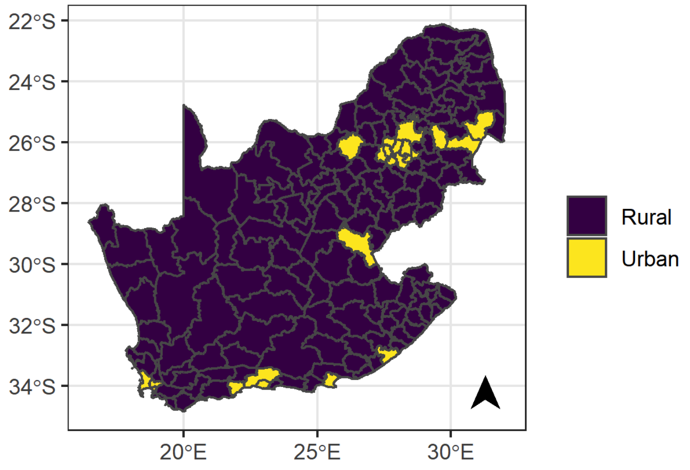

Figure S3: The number of health facilities by municipality and year (left panel) and by municipality in 2014 (right panel).

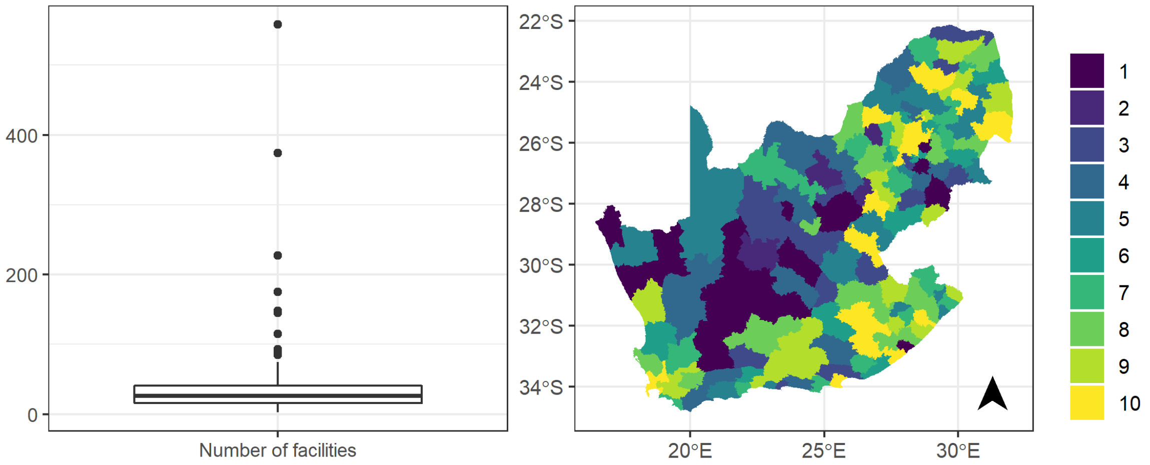

Figure S4: The spatial variation of socioeconomic index in South Africa. A rank of 1 denotes the most deprived area.

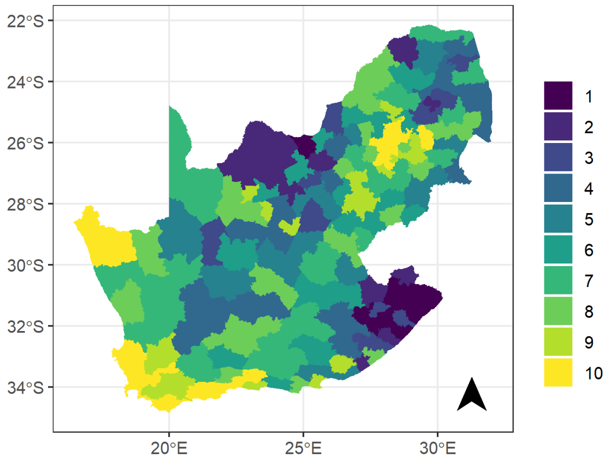

Figure S5: Provinces in South Africa in 2016 [ROSEA].

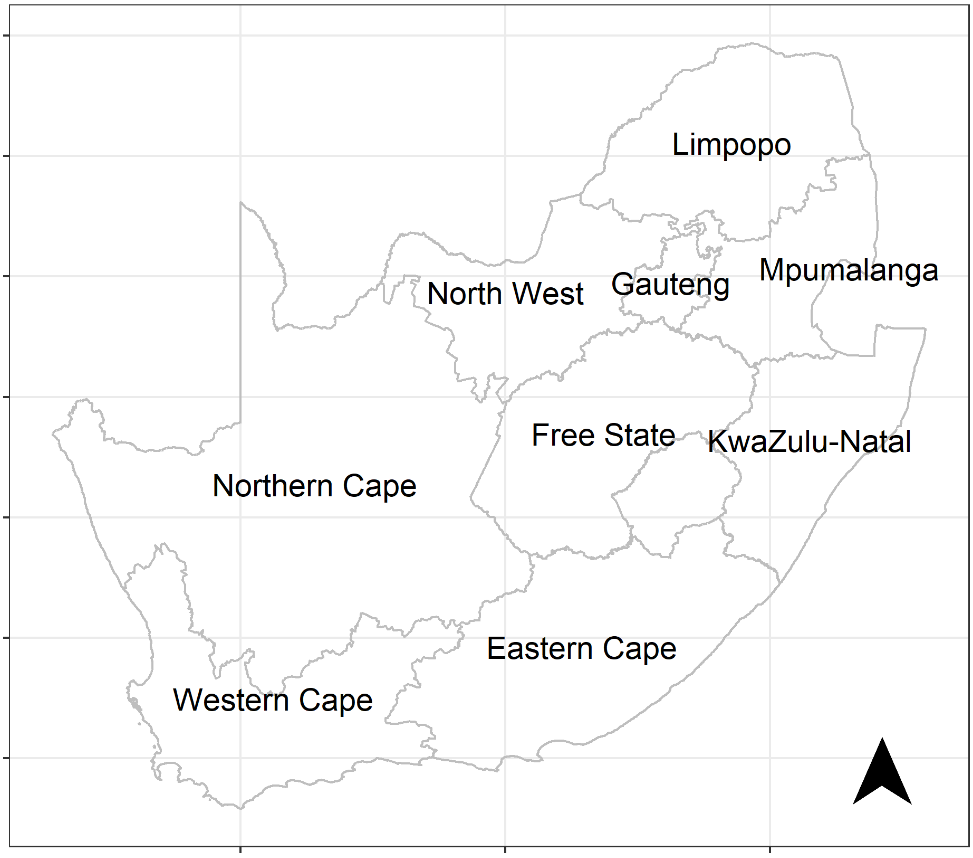

Figure S6: Municipalities in South Africa in 2016 [ROSEA].

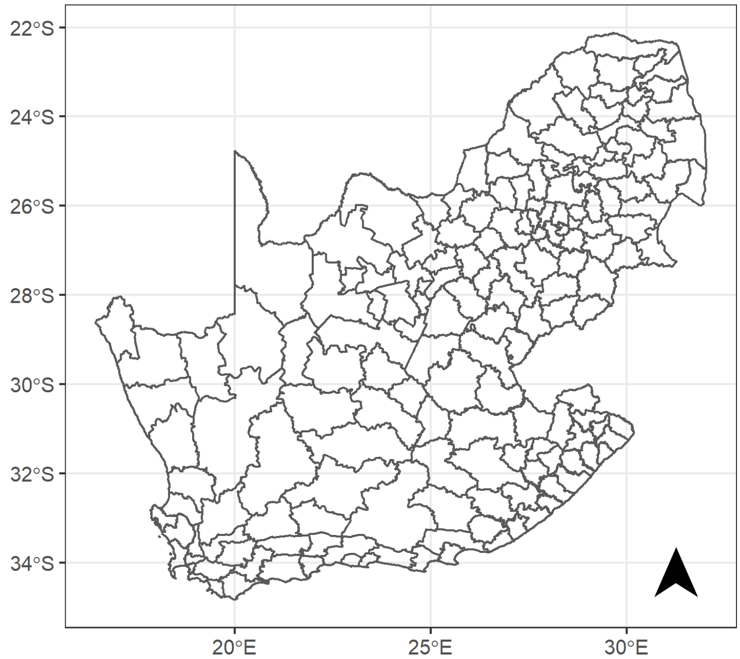

Figure S7: Median posterior of spatial relative risk (exponential of the spatial random effect) and posterior probability using correction I, II and full correction.

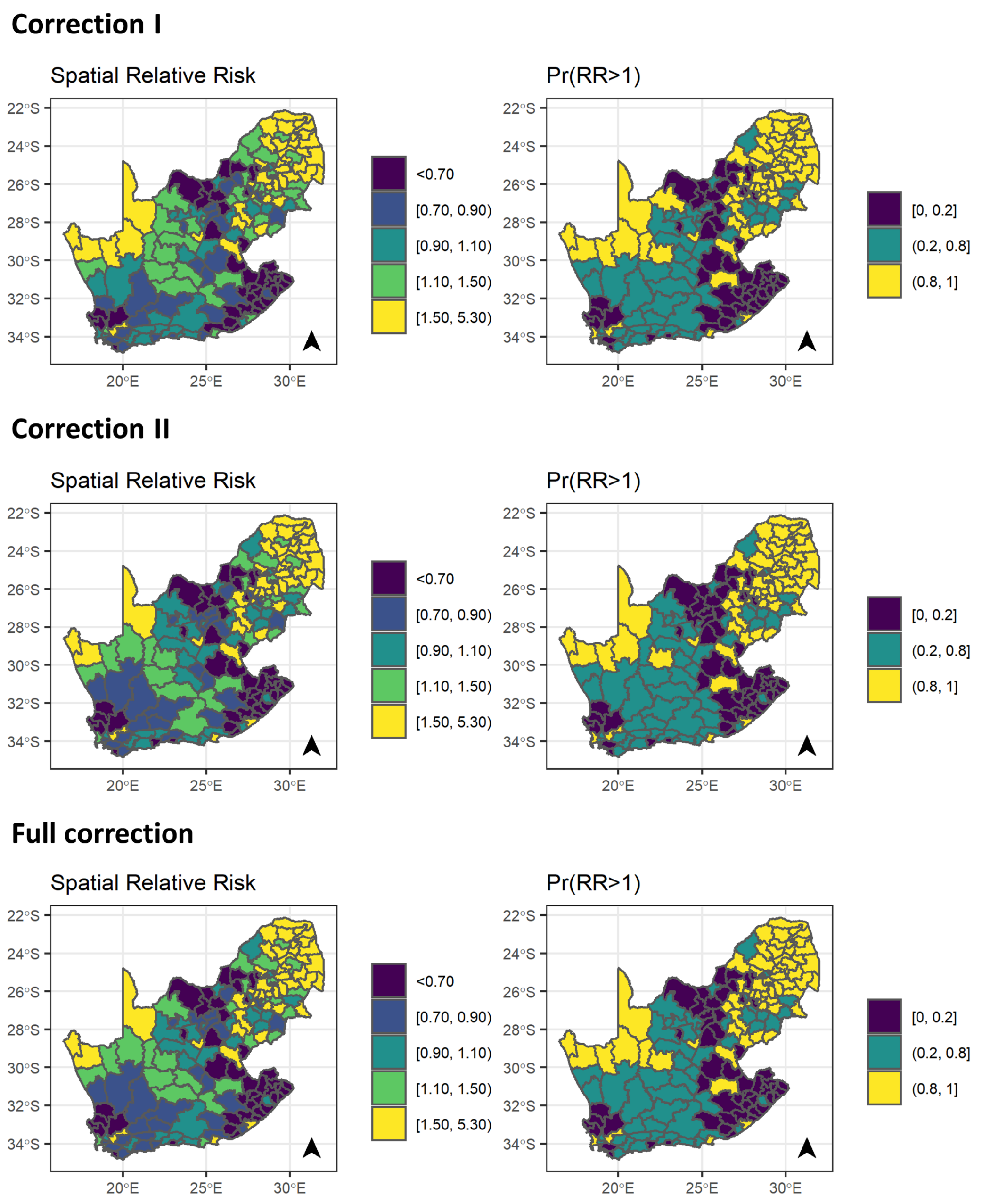

Figure S8: Posterior probability that the spatiotemporal relative risk (relative to the national average over time) is higher than 1 of cervical cancers among women living with HIV in South Africa, using the no correction model adjusted for the selected covariates.

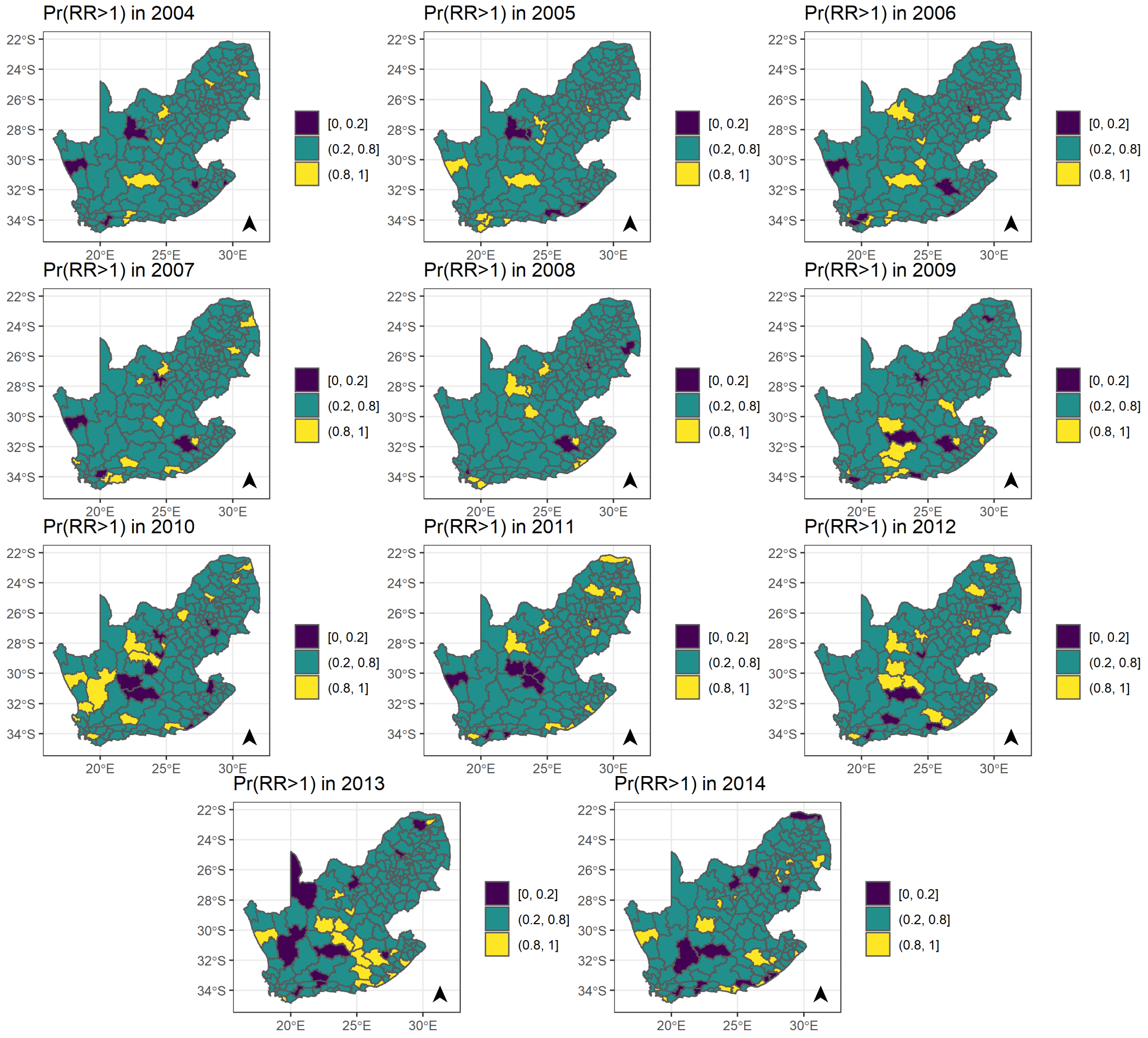

Figure S9: Median posterior of spatial relative risk (exponential of the spatial random effect) of cervical cancers compared to the national average during 2004-2014 for the model without any covariates.

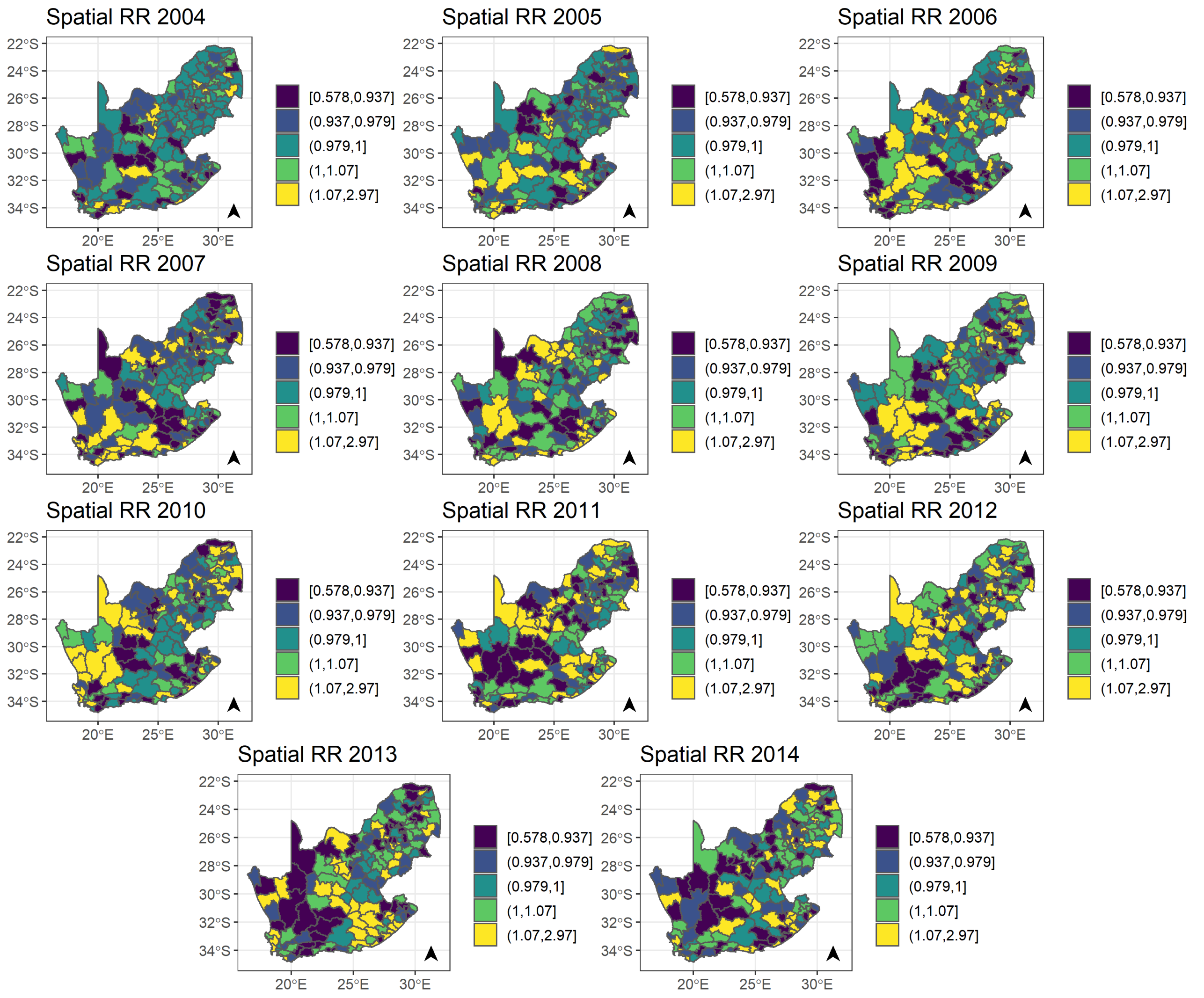

Figure S10: Median posterior of spatial relative risk (exponential of the spatial random effect) of cervical cancers compared to the national average during 2004-2014 for the model without any covariates and using correction I.

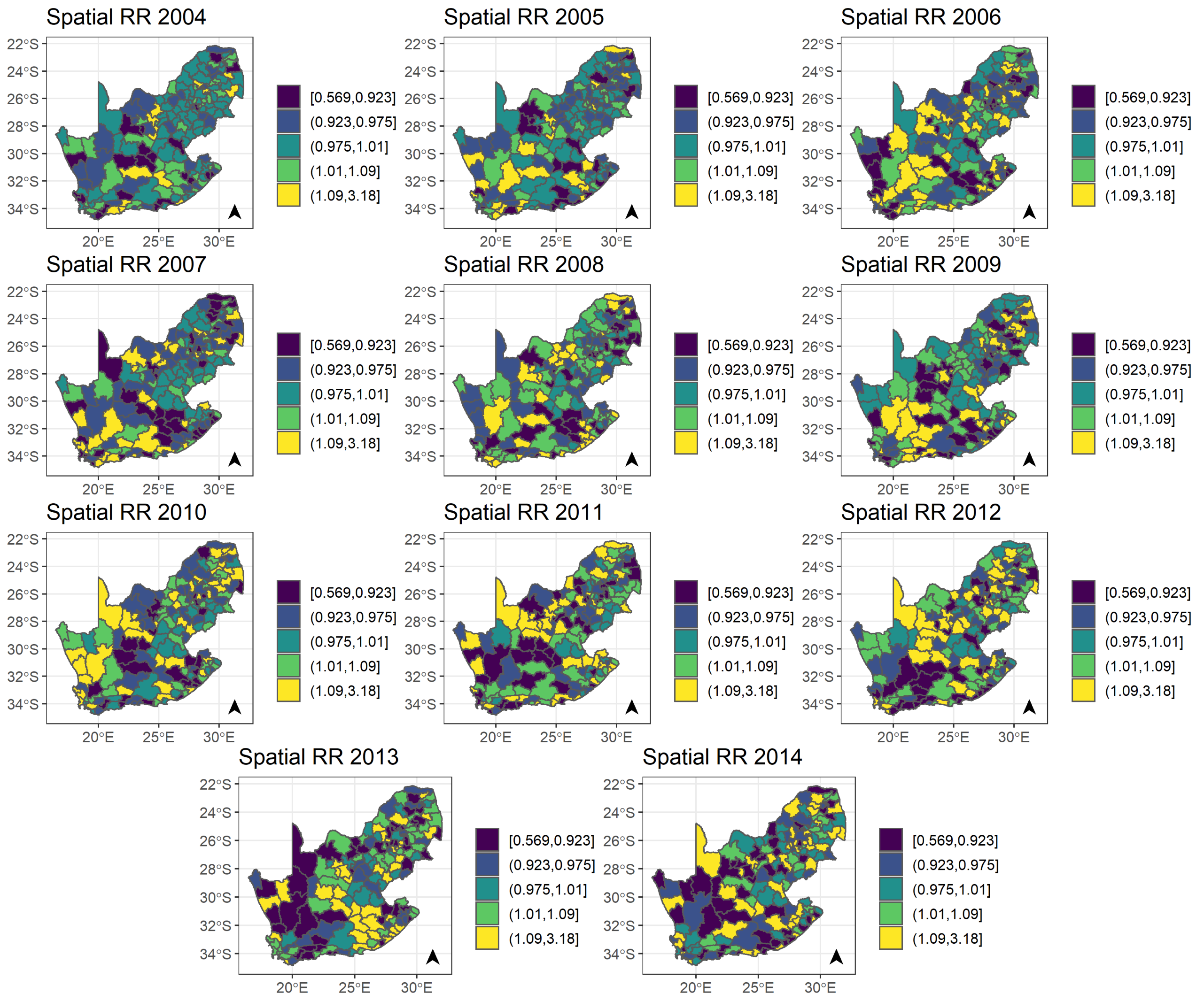

Figure S11: Median posterior of spatial relative risk (exponential of the spatial random effect) of cervical cancers compared to the national average during 2004-2014 for the model without any covariates and using correction II.

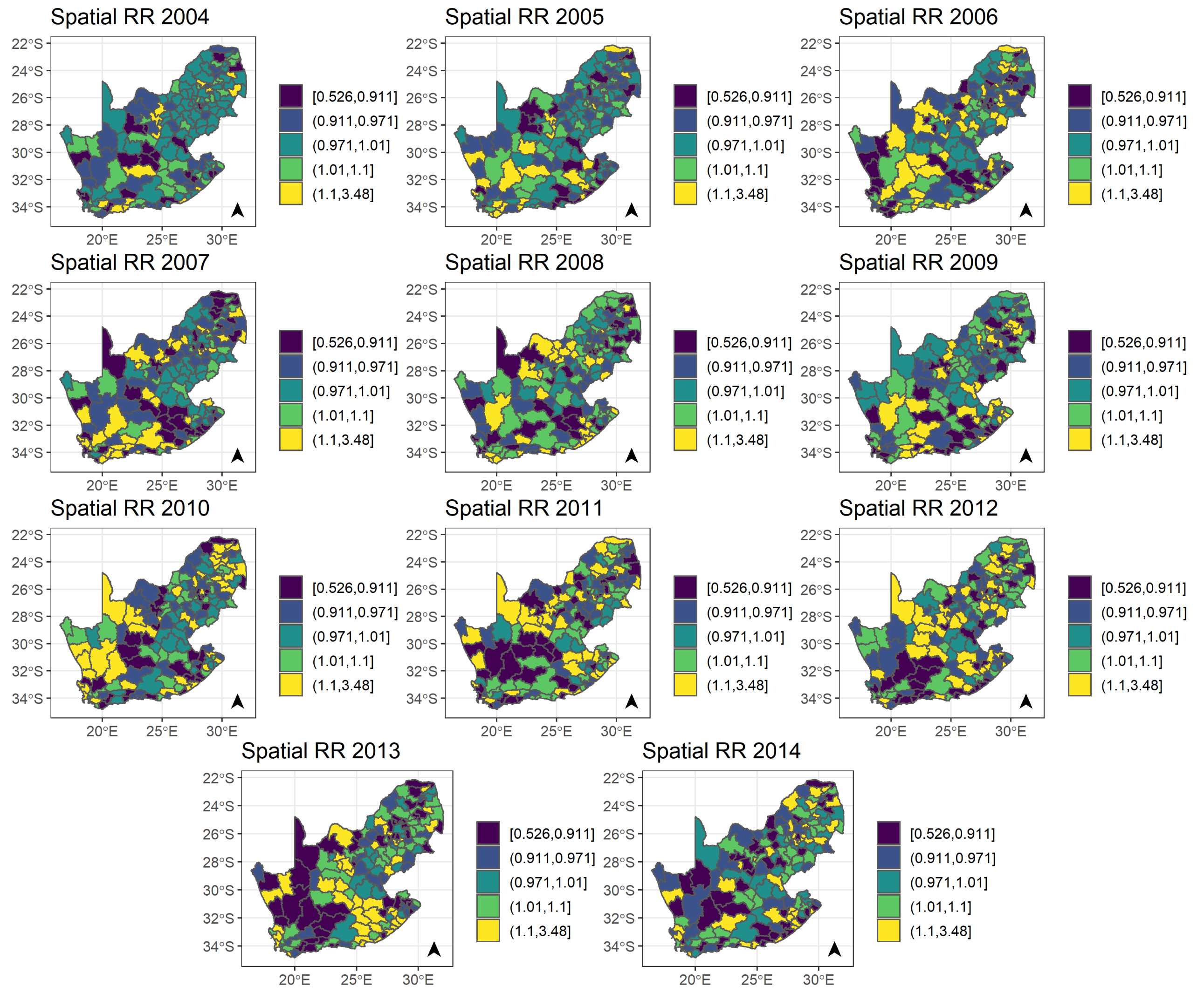

Figure S12: Median posterior of spatial relative risk (exponential of the spatial random effect) of cervical cancers compared to the national average during 2004-2014 for the model without any covariates and using the full correction.

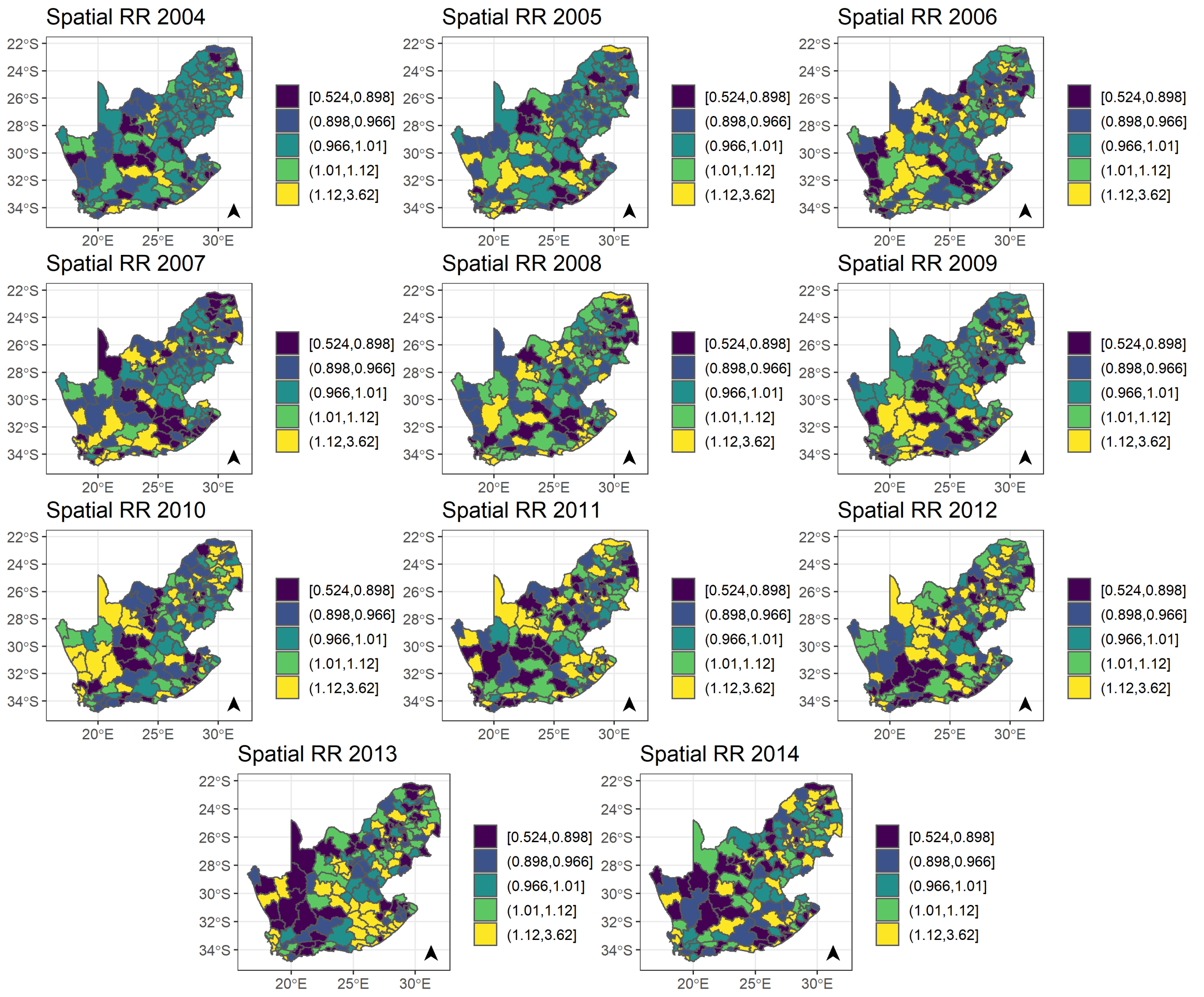

Figure S13: Posterior probability that the spatiotemporal relative risk (relative to the national average over time) is higher than 1 of cervical cancers among women living with HIV in South Africa, using correction I and the model without any covariates.

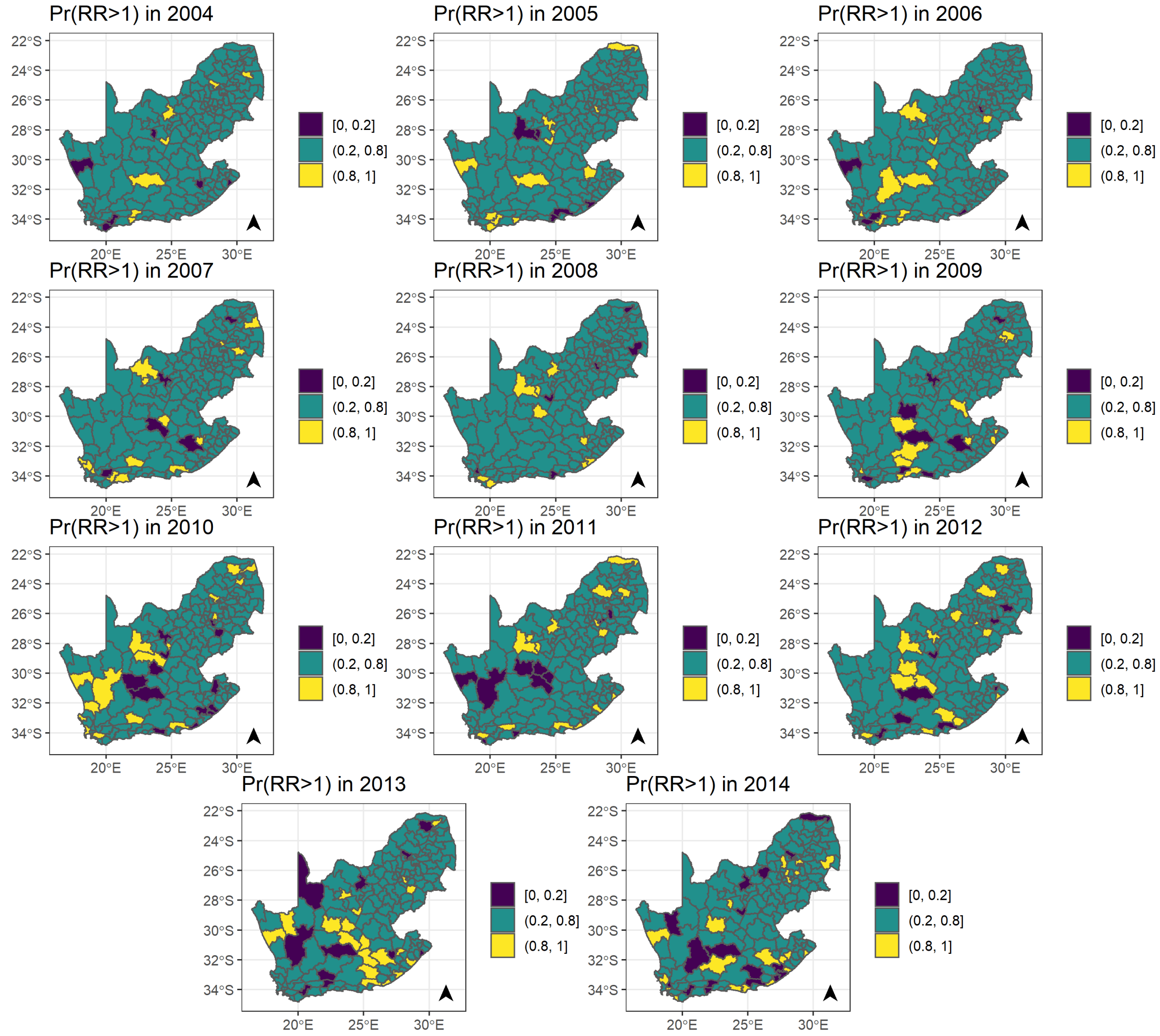

Figure S14: Posterior probability that the spatiotemporal relative risk (relative to the national average over time) is higher than 1 of cervical cancers among women living with HIV in South Africa, using correction II and the model without any covariates.

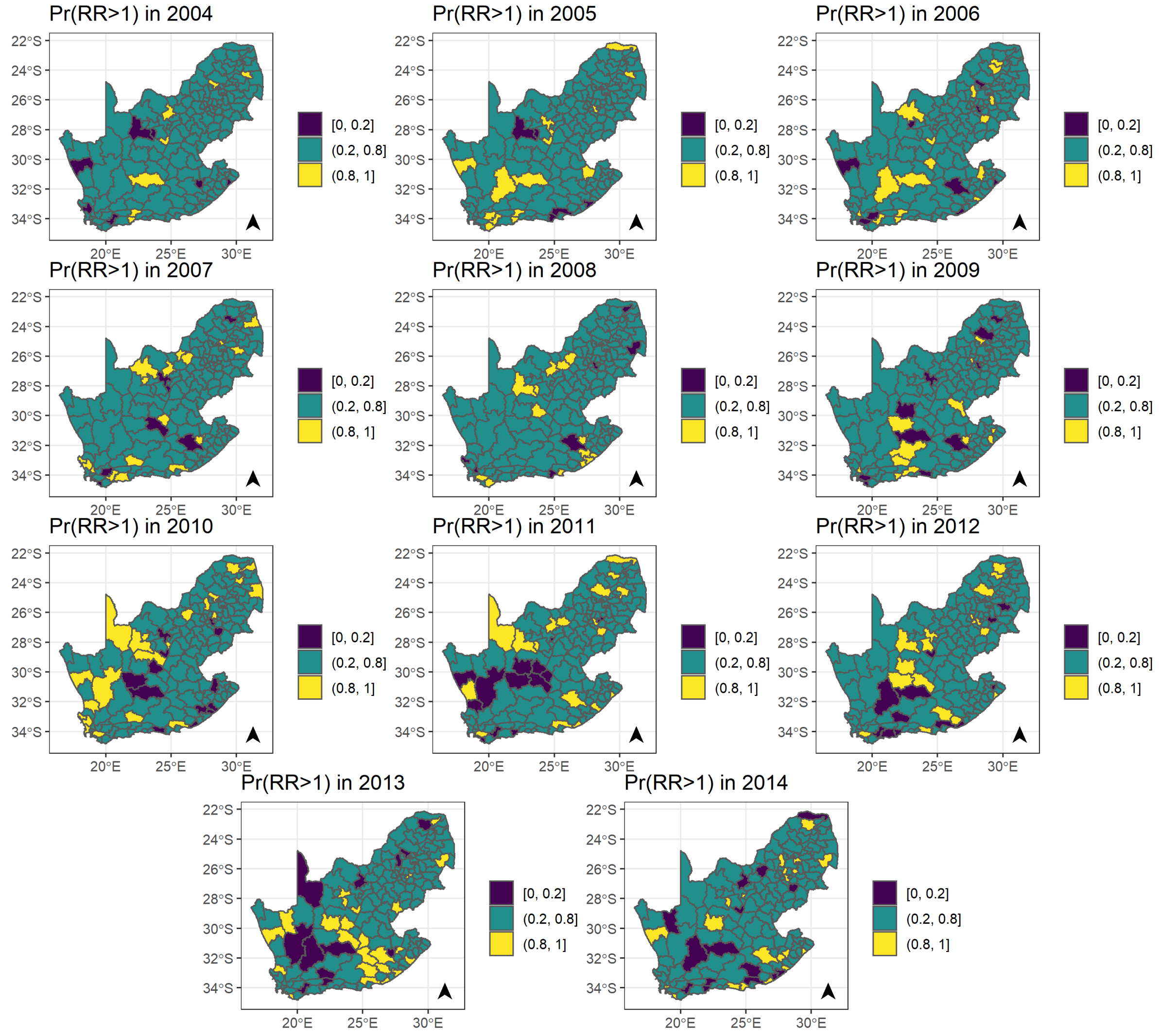

Figure S15: Posterior probability that the spatiotemporal relative risk (relative to the national average over time) is higher than 1 of cervical cancers among women living with HIV in South Africa, using full correction and the model without any covariates.

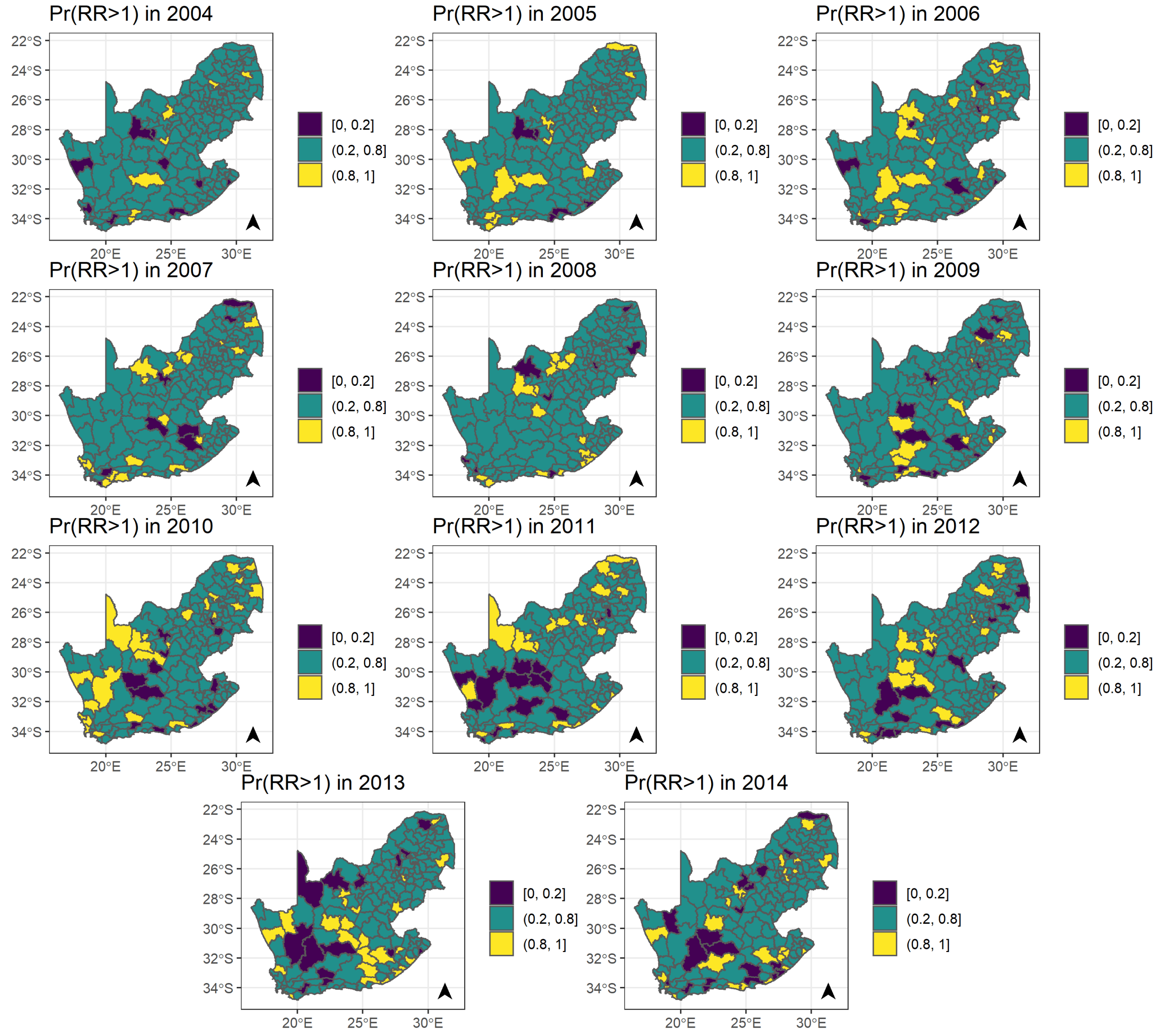

References

Julian Besag, Jeremy York, and Annie Mollié. Bayesian image restoration, with two applications in spatial statistics. *Annals of the institute of statistical mathematics*, 43(1):1–20, 1991.

Leonhard Knorr-Held. Bayesian modelling of inseparable space-time variation in disease risk. *Statistics in medicine*, 19(17-18):2555–2567, 2000.

OCHA ROSEA. Subnational administrative boundaries. URL `https://data.humdata.org/dataset/south-africa-admin-level-1-boundaries`.

Daniel Simpson, Håvard Rue, Andrea Riebler, Thiago G Martins, Sigrunn H Sørbye, et al. Penalising model component complexity: A principled, practical approach to constructing priors. *Statistical science*, 32(1):1–28, 2017.

Table S2: Annual median and 95% Credibility intervals (CrI) for the incidence rate of cervical cancers among HIV positive women per 100,000 person years in South Africa for the different corrections considered.

	No correction		Correction I		Correction II		Full Correction	
year	Median	95% CrI	Median	95% CrI	Median	95% CrI	Median	95% CrI
2004	306	(169, 555)	306	(163, 573)	310	(164, 589)	312	(160, 609)
2005	386	(221, 675)	378	(211, 680)	398	(224, 718)	394	(217, 727)
2006	417	(243, 718)	407	(231, 720)	436	(249, 771)	430	(241, 775)
2007	341	(198, 588)	336	(190, 593)	357	(203, 630)	354	(198, 636)
2008	319	(187, 543)	318	(183, 553)	340	(197, 590)	341	(195, 603)
2009	294	(174, 498)	297	(172, 514)	314	(183, 542)	319	(183, 559)
2010	269	(160, 451)	272	(159, 467)	289	(170, 494)	294	(171, 510)
2011	231	(139, 386)	237	(140, 403)	249	(149, 422)	256	(151, 440)
2012	203	(122, 338)	209	(123, 353)	218	(130, 367)	224	(132, 383)
2013	179	(108, 296)	187	(111, 316)	193	(116, 324)	202	(120, 344)
2014	160	(96, 265)	179	(106, 303)	172	(103, 290)	191	(113, 326)
Median	294	(174, 498)	297	(163, 573)	310	(170, 494)	312	(171, 510)

Table S3: Results of the model with spatial, temporal and spatiotemporal interaction and deprivation and urbanicity for the different Thembisa denominators.

	Univariable		Multivariable	
	median	95% CrI	median	95% CrI
$1/\sigma_1^{2*}$	32.36	(10.93, 83.24)	33.05	(11.88, 83.25)
$1/\sigma_2^{2*}$	3.53	(2.52, 4.84)	4.07	(2.75, 5.94)
ρ	0.62	(0.36, 0.84)	0.77	(0.45, 0.95)
$1/\sigma_3^{2*}$	1.28	(0.64, 2.41)	1.28	(0.64, 2.43)
$1/\sigma_4^{2*}$	16.22	(12.82, 20.69)	16.05	(12.69, 20.41)
